# Structural and functional characterisation of the dextran utilisome from *Bacteroides thetaiotaomicron*

**DOI:** 10.1016/j.yjsbx.2026.100153

**Published:** 2026-07-17

**Authors:** Matthew Feasey, Augustinas Silale, Arnaud Baslé, Bert van den Berg

**Affiliations:** Biosciences Institute, The Medical School, Newcastle University, Newcastle upon Tyne NE2 4HH, UK

**Keywords:** Gut *Bacteroides*, Glycan processing and uptake, Utilisome, Cryo-EM, X-ray crystallography

## Abstract

*Bacteroides thetaiotaomicron* (*B. theta*) is a model Bacteroidota of the healthy human gut microbiota and a specialist in glycan utilisation. Like other *Bacteroides*, *B. theta* has many highly regulated polysaccharide utilisation loci (PUL) that encode outer membrane (OM) TonB-dependent transporters (SusC), closely associated “lid” lipoproteins (SusD), and additional surface-exposed lipoproteins (SLPs) that bind and partially degrade specific glycans derived from host cells, diet, or other microbiota members. The canonical starch PUL products are thought to form a dynamic complex in the presence of starch. However, other PULs form stable complexes in the absence of substrate (recently named “utilisomes”), with additional surface lipoproteins tightly associated with the core SusCD complex. In this study, we characterised the *B. theta* dextran utilisome, with a SusCD^dex^ core and an associated glycoside hydrolase (GH^dex^) and surface glycan binding protein (SBGP^dex^). Via X-ray crystallography we solved high-resolution structures of SBGP^dex^ in isolation and SusD^dex^ and GH^dex^ bound to dextran oligosaccharides. We used isothermal titration calorimetry (ITC) to quantify ligand binding of wild type and mutant SLPs. We further used single particle cryo-EM of the catalytically inactive dextran utilisome to visualise open and closed states of the complex. Three occupied dextran binding sites were observed across SusC^dex^, SusD^dex^ and GH^dex^, with substrate observed in both open and closed states of SusD^dex^. 3D variability analysis showed a minority of particles in the process of SusD^dex^ lid closure. Together our work defines commonalities and differences across utilisomes dedicated to the import of simple glycans.

## Highlights

• Utilisomes are multicomponent protein complexes on the cell surface of gut *Bacteroides*.
• Utilisomes are nanoscale processing plants that mediate the coordinated breakdown and uptake of glycans such as dextran.
• We present a structural and functional characterisation of the *Bacteroides thetaiotaomicron* dextran utilisome.
• Dextran oligosaccharide binding sites are identified by X-ray crystallography, cryo-EM and isothermal titration calorimetry.
• Cryo-EM reveals utilisome dynamics as well as differences and similarities to the previously characterised levan utilisome.

## Graphical abstract

**Figure:**
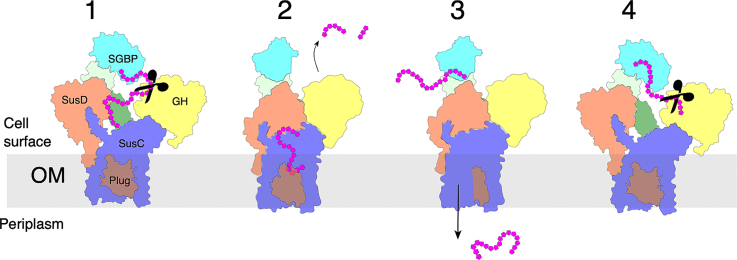

## 1 Introduction

The human gastrointestinal (GI) tract is home to a wide array of microbes, collectively termed the human gut microbiota (HGM). Intense research efforts over the past 2 decades have revealed that this bacteria-dominated community has far-reaching effects on human health. Complex dietary glycans (“fibre”) that cannot be degraded by enzymes of the human digestive tract are thought to be the primary nutrient source for the HGM, and the utilisation of these complex sugars is crucial for the relationship between host and bacteria, leading, for example, to the generation of short-chain fatty acids that provide metabolic energy and systemic health benefits to the host. The colon or large intestine is the most densely populated part of the GI tract, with microbial densities up to 10^11^–10^12^ per gram of luminal content (Sasso et al., 2023). Most of the colonic microbiota belongs to two bacterial phyla: the Gram-positive Bacillota and the Gram-negative Bacteroidota. Additional, minor phyla include Actinomycetota and Pseudomonadota (Sasso et al., 2023; MetaHIT Consortium et al., 2010; Grondin et al., 2017).

Belonging to the Bacteroidota, members of the genus *Bacteroides* have attracted particular attention for several reasons. First, several *Bacteroides* species (*B. fragilis*, *B. thetaiotaomicron*) (Comstock and Coyne, 2003; Zocco et al., 2007) are relatively easy to culture in the lab and can be manipulated genetically. Second, the *Bacteroides* are collectively the most abundant bacterial genus in the gut of western world populations, comprising up to 40% of all bacteria in the large intestine (MetaHIT Consortium (additional members) et al., 2011). As such, *Bacteroides* play an important role in shaping the HGM, and their study can be expected to yield important discoveries on fundamental aspects of the gut microbial ecosystem. Third, Bacteroides are prolific, primary degraders of many dietary glycans, producing many oligosaccharides that can be used by other members of the HGM, a phenomenon called cross-feeding. The best-studied member of the *Bacteroides*, *B. thetaiotaomicron* VPI-5482 *(B. theta)*, is considered a glycan “generalist” owing to the ∼18% of its 6.2 Mb genome that is dedicated to the utilisation of glycans (Grondin et al., 2017; Xu et al., 2003).

The *co*-transcribed genes involved in the degradation of a particular glycan are organised on the genome in so-called PULs (polysaccharide utilisation loci). According to the PUL database (http://www.cazy.org/PULDB/), *B. theta* has 86 predicted PULs, and for most of these no ligands have been identified experimentally. PUL prediction is based on the presence of at least one SusCD pair, consisting of a SusC (from starch utilisation system or saccharide utilisation system) TonB-dependent transporter (TBDT) and a tightly associated SusD ligand binding protein. SusDs are surface-exposed lipoproteins (SLPs) that form a mobile cap on the SusC transporter to deliver transport-competent substrates to the SusC via a “pedal-bin” mechanism (Glenwright et al., 2017; Gray et al., 2021; White et al., 2023). In addition to SusD proteins, PULs typically include additional SLPs such as surface glycan binding proteins (SGBPs) and glycoside hydrolases (GHs), involved in the initial binding of polymeric glycans and degradation of those glycans into transport-competent oligosaccharides, respectively (Foley et al., 2016; Terrapon et al., 2018). It should be noted that approximately a quarter of predicted PULs do not contain proteins with obvious glycan binding or degradation functionalities, suggesting that non-glycan molecules such as peptides (Glenwright et al., 2017) are also taken up by “PULs”.

Recently it was shown that for the “simple” glycan levan (a β2,6-linked fructan), all four outer membrane (OM) components (BT1760–3) of the respective PUL form large (∼600 kDa) and stable complexes on the cell surface, irrespective of bound glycans, that were named “utilisomes” (White et al., 2023). Cryo-EM structures of the purified utilisome revealed a dimer of tetramers each consisting of a GH^lev^, SGBP^lev^, SusD^lev^ and SusC^lev^, and the effect of levan substrates on the dynamics of the catalytically inactivated utilisome was determined. In the same study, a cryo-EM structure of the wild-type, apo dextran utilisome was also reported (White et al., 2023) that showed a similar architecture as the levan utilisome, but with no information on substrate binding by any of the components. The dextran PUL (PUL48) is known to be upregulated in the presence of dextran (White et al., 2023; Jones et al., 2019; Wong et al., 2024; Rogers et al., 2013).

Here, PUL48 was further characterised. We determined X-ray crystal structures of *E. coli*-expressed soluble variants of GH^dex^, SGBP^dex^, and SusD^dex^ without (SGBP^dex^) and with (GH^dex^ and SusD^dex^) bound dextran-derived isomaltooligosaccharides (IMO), allowing the identification of important residues in GH^dex^ and SusD^dex^ for dextran binding. The affinities of the three proteins (wild type and binding mutants) for seven differently sized dextran substrates (from 1.5 to 500 kDa) were measured using ITC. For both GH^dex^ and SusD^dex^, the affinities for dextran substrates are in the low μM range and roughly independent of dextran length. By contrast, the affinity of SGBP^dex^ for dextran substrates increases greatly with increasing ligand size to reach a similar affinity as GH^dex^ and SusD^dex^ for the longest dextran tested (500 kDa). The entire catalytically inactivated dextran utilisome was purified from *B. theta* cells via His-tagged SusD^dex^. Cryo-EM structures were determined in the presence of dextran substrate, revealing both similarities and differences compared to the levan utilisome that increase our understanding of glycan uptake by gut *Bacteroides*.

## 2 Results

### 2.1 Expression and purification of the dextran SLPs GH^dex^, SGBP^dex^ and SusD^dex^ from *E. coli.*

**Fig. 1:**
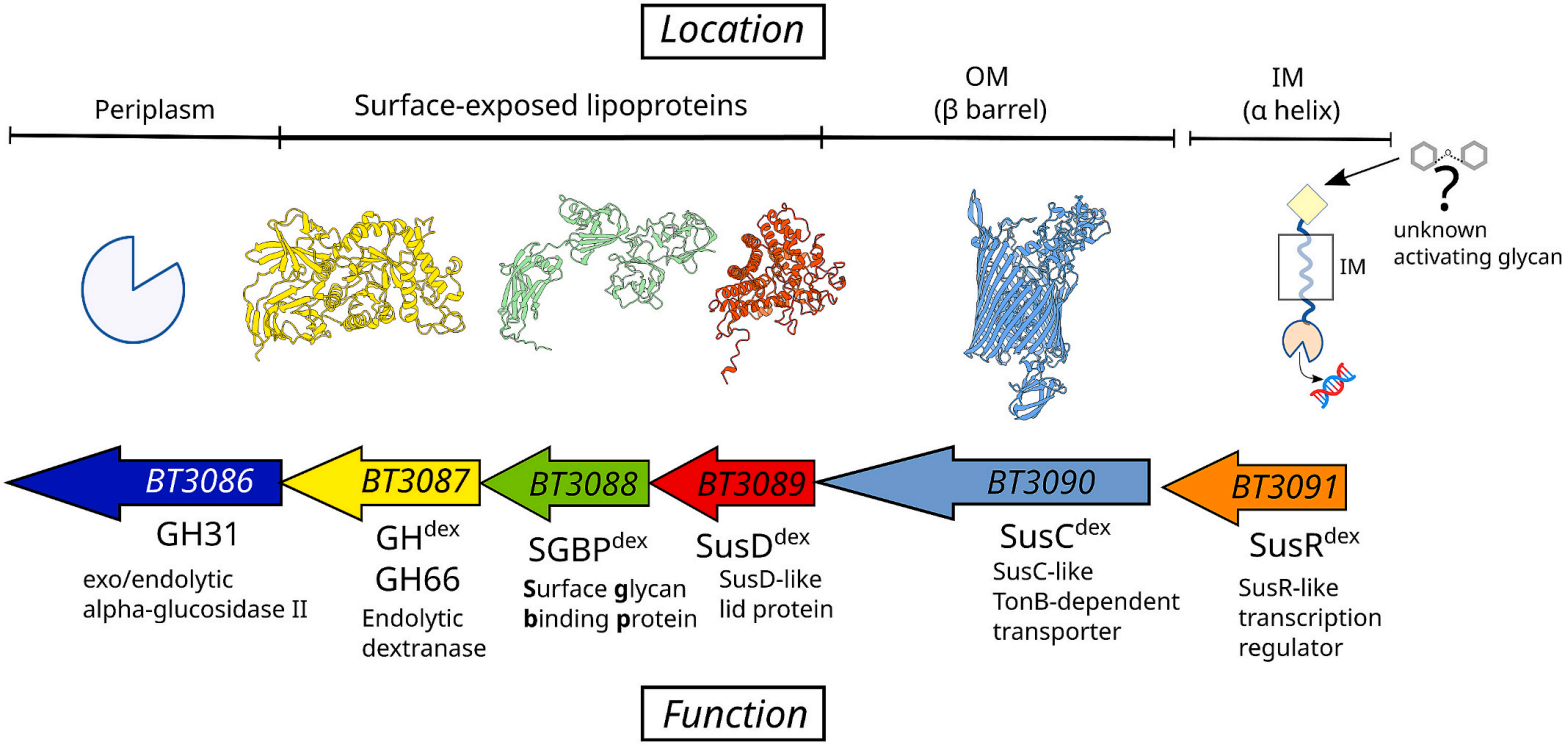
The dextran PUL of *B. theta* (PUL48). The dextran PUL and its constituent proteins, including Alphafold2 (Jumper et al., 2021) models of the individual outer membrane-associated components.

For further analysis of the OM components of PUL48, GH^dex^, SGBP^dex^ and SusD^dex^ (*bt3087*, *bt3088* and *bt3089* respectively; Fig. 1A), we generated *E. coli* constructs in which the cysteine that would be lipidated in the mature lipoprotein was removed. In all three SLPs, an additional 9 N-terminal residues, predicted by AlphaFold2 (Jumper et al., 2021) to be unstructured, were also removed (Methods). This allowed high-yield overexpression of the PUL48 SLPs as soluble proteins (Fig. S1). These proteins were then purified (Fig. 1B) for X-ray crystallography and ITC. Average protein yields of the GH^dex^ constructs, including the catalytic mutants, was approximately 3 mg protein per litre of culture. Yields for SGBP^dex^ and SusD^dex^ were 7 and 5 mg/L, respectively.

### 2.2 Structure determination of GH^dex^ in the absence and presence of substrate.

GH^dex^ is a GH66 family *endo*-dextranase that hydrolyses the α1,6 backbone of dextran (Kim et al., 2012a). A study previously identified its two putative conserved catalytic residues, Asp297 and Glu360, by homology analysis between GH66 dextranases and chemical rescue experiments of *Paenibacillus* dextranase (Kim et al., 2012a; Kim et al., 2012b). Homologous residues are also found in dextranases from *Streptococcus mutans* INGBRITT and *Bacillus circulans* T-3040 (Kim et al., 2012a). In the retaining mechanism of hydrolysis, the Asp carboxylate acts as a nucleophile while the Glu acts as an acid/base (Kim et al., 2012a; Pengthaisong et al., 2023). To verify the effect of the proposed GH^dex^ catalytic pair, we generated GH^dex^ pET28b constructs for D297A and E360A, as well as a double mutant D297A/E360A (GH^dex⁎^). These were then expressed as soluble proteins in *E. coli*. Since we assumed that GH^dex⁎^ would be completely inactive, we tested the E360A mutant for its ability to digest dextran 500 in vitro (Fig. S2). WT GH^dex^ generated a ladder of glycans over the course of the reaction, culminating in glycans with a degree of polymerisation (DP) of 1–3. GH^dex^ E360A generated no degradation products, but the intensity of the unmigrated dextran 500 spots decreased with increasing incubation times, suggesting some residual activity.

**Fig. 2:**
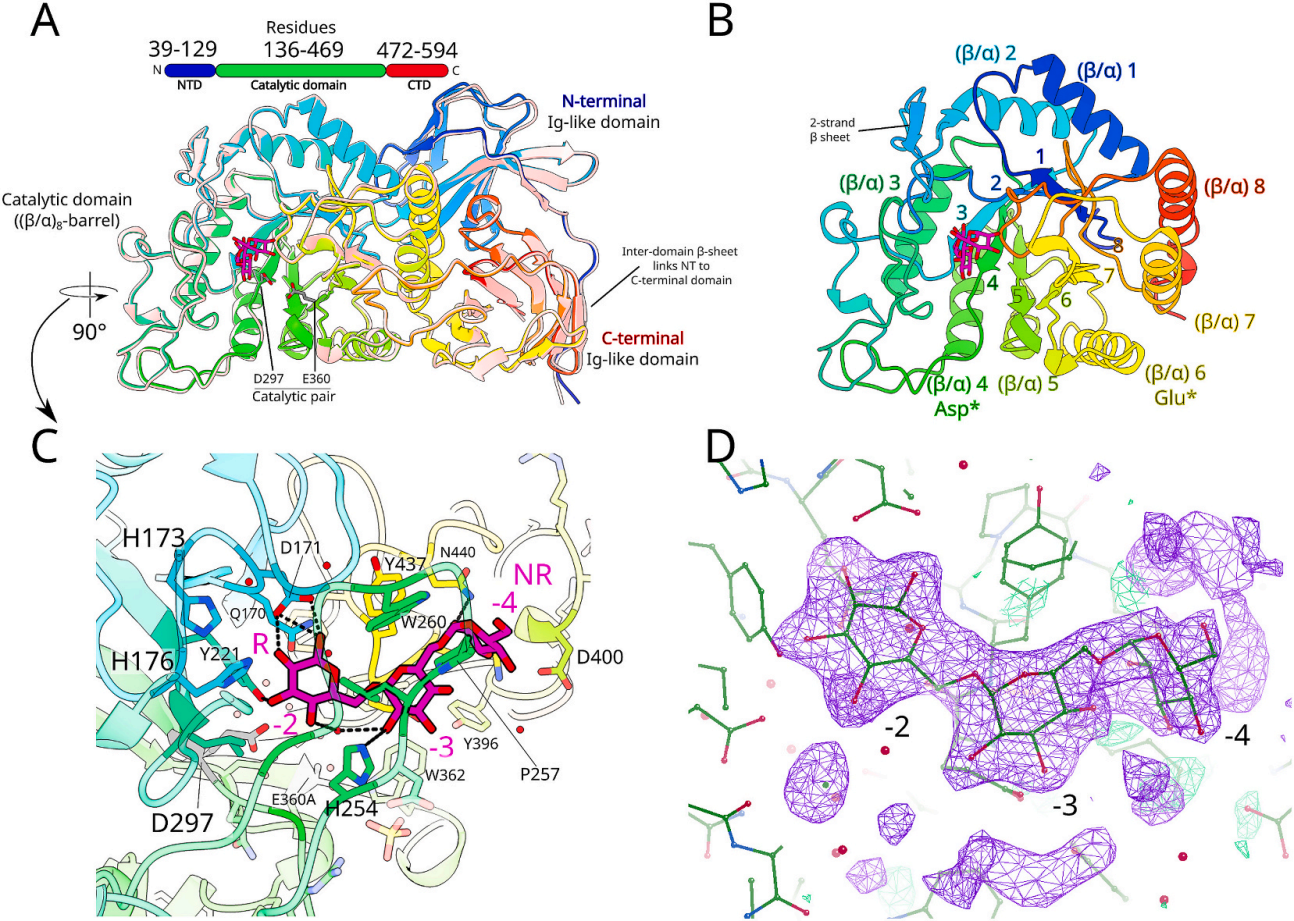
The crystal structures of wild-type (WT) GH^dex^ and the catalytic mutant GH^dex^ E360A. A. Overlaid cartoon displays of apo WT GH^dex^ (in salmon) and the E360A catalytic inactive mutant (in rainbow) with bound isomaltotriose. Domain assignment is shown for the primary sequence at top, coloured by domain (N-terminal domain (NTD) is in blue, the catalytic domain is in green, and the C-terminal domain (CTD) is in red). In the WT structure the side chains of the catalytic residues D297 and E360 are displayed in grey. B. The (β-α)_8_ catalytic domain of GH^dex^ E360A is shown and recoloured in rainbow format. β-strands that contain the catalytic residues are marked with an asterisk. C. The binding site of GH^dex^ E360A, chain B. Putative subsites for each glucose residue are numbered, with non-reducing (NR) and reducing (R) ends labelled. Residues were first numbered (with increasingly negative numbers from the catalytic site to the NR end) based on homology with *Tp*^dex^ (Suzuki et al., 2016) (Fig. S3) and then confirmed in the cryo-EM structure of GH^dex^ (Fig. 6). Residues <5 Å from the isomaltotriose ligand are highlighted in full colour, with side chains displayed for residues with hydrogen bonds to the ligand (represented by dashed lines), residues that could hydrogen bond with a longer ligand, and aromatic residues likely important for binding. The catalytic residues or their substituted residue (D297, E360A) are coloured in grey. D. The modelled isomaltotriose is shown in a Polder map (Liebschner et al., 2017) calculated by *Phenix* contoured at 3 σ in *Coot*. Structures of apo GH^dex^ (WT) and GH^dex^ E360A with and without isomaltotriose were deposited under PDB IDs 9SJG, 9SJF and 9SJH, respectively. (For interpretation of the references to colour in this figure legend, the reader is referred to the web version of this article.)

The X-ray structure of wild-type GH^dex^ was solved to a resolution of 1.8 Å (PDB ID: 9SJG), with apo GH^dex^ E360A solved to 2.1 Å (PDB ID: 9SJH) and the same mutant co-crystallised with dextran 1.5 was solved to 2.2 Å (PDB ID: 9SJF) (see Fig. 2, and Table S1 for X-ray crystallography statistics). GH^dex^ contains three domains. The N-terminal and C -terminal domains (NTD, CTD) contain Ig-like folds with 8 and 11 β-strands, respectively. Preceding the NTD, a short β-strand (residues 31–33) forms an inter-domain β-sheet with the CTD, which may rigidify these domains. The catalytic domain consists of a (β/α)_8_ barrel (residues 136–469, Fig. 2B), and the catalytic pair D297 and E360 (Suzuki et al., 2012; Suzuki et al., 2016) is found within the catalytic domain cavity on the 4th and 6th β-strands of the barrel. The catalytic domain forms a pocket or crater-like topology, which has been previously described in glycoside hydrolases that hydrolyse exopolysaccharides (Davies and Henrissat, 1995). The distance between the catalytic carboxylates is 6.5 Å, similar to the reported distance in retaining mechanism enzymes (Pengthaisong et al., 2023; Davies and Henrissat, 1995).

As expected, GH^dex^ is divergent from GH^lev^ (GH32) and the canonical SusG (GH^starch^, GH13) (White et al., 2023; Koropatkin and Smith, 2010), with Cα RMSD values of 14.3 Å and 48.5 Å, respectively (Fig. S4). Comparison with the GH^dex^ Alphafold prediction indicated minimal differences with a Cα RMSD of 0.6 Å. When GH^dex^ was compared to *Thermoanaerobacter pseudoethanolicus* dextranase (*Tp*^dex^, PDB ID: 5AXG, 5AXH (Suzuki et al., 2016), see also Fig. S3 and the Supplementary Discussion), the structures were found to be highly similar with Cα RMSD of 3.2 Å, or 1.0 Å when only the catalytic domains were compared. In the mutant *Tp*^dex^ structure liganded with isomaltohexaose, the authors replaced the catalytic nucleophile (D312G), and the bound ligand spanned subsites −4 to +2. Comparison with GH^dex^ E360A showed that the ligand conformations were highly similar, with three glucose residues of dextran 1.5 resolved above the active site (subsites −2 to −4), equivalent to isomaltotriose (IMO3). This suggests that the E360A mutant retained some catalytic activity, cleaving dextran on the timescale of crystallisation (several days, 20 °C, pH 5.6). This agrees with the dextran digestion assay, in which the total amount of dextran incubated with GH^dex^ E360A decreased during the assay (Fig. S2). Taken together, these results suggest the aspartate nucleophile of GH^dex^ is more important for catalysis than the glutamate acid/base, which is reflected in the literature for other retaining glycoside hydrolases. In the retaining mechanism, nucleophile mutants are unable to form the covalent glycosyl-enzyme intermediate and therefore exhibit a dramatic loss of activity that is generally less recoverable under standard chemical rescue conditions. By contrast, acid/base mutants may retain low levels of residual activity and can sometimes be partially rescued by exogenous proton donors. For example, two previous studies on a retaining cyclodextrin glycosyltransferase reported a much larger decrease in activity when the catalytic nucleophile Asp was substituted compared to the acid/base Glu (Mosi et al., 1997; Wind et al., 1998; Ly and Withers, 1999). The double mutant (GH^dex⁎^) was also subjected to crystallisation screens, and needle-like crystals formed in similar conditions (Materials & Methods). However, sufficiently large crystals for data collection were not obtained after optimisation.

If catalysis is still occurring in GH^dex^ E360A, it is puzzling why ligand was not resolved at subsite −1 (see Fig. 2). Although GH^dex^ E360A with dextran did not yield any observable product in the dextran digestion assay (Fig. S2), the wild-type showed accumulation of predominantly DP1–3 products, possibly suggesting that the IMO3 substrate was the result of fractional catalytic activity retained in GH^dex^ E360A. Additionally, the crystal structure did not capture a catalytically aligned substrate or product state. Instead, ligand density is observed predominantly in the −2 to −4 subsites, with weak density extending beyond the non-reducing terminus of the −4 subsite in the Polder map (Fig. 2D), and a clear absence of density at the −1 subsite. This indicates that the crystal structure represents a non-productive binding mode involving distal subsite interactions, while the catalytic centre is not engaged. The absence of occupancy in subsite −1 is therefore unlikely to be solely due to local disorder, but rather reflects a preferred binding mode that avoids the catalytic −1 pocket in the crystallisation conditions. Taken together with the digestion assay, these results suggest that IMO3 may represent a relatively stable hydrolysis product of GH^dex^ that preferentially associates with distal subsites (−2 to −4). The preference of IMO3 for distal subsites would likely reduce engagement of the catalytic centre, contributing to its persistence in the digestion assay, which is consistent with the non-productive binding mode in the crystal structure. It is worth noting that the dextran substrate is almost identical to a crystal structure of a triose bound to the *Flavobacterium johnsoniae* GH66 dextranase, which is the most similar structural homologue in the PDB (with ∼48% sequence identity to GH^dex^) (Nakamura et al., 2023). The authors of the *Tp*^dex^ crystal structure noted that between the two molecules of the asymmetric unit, Trp376 either occluded subsite +1 or provided a “side wall” of the catalytic cleft, and this may be responsible for the expulsion of hydrolysed glycans (Fig. S3E—F) (Suzuki et al., 2016). However, this was not observed in crystal or cryo-EM structures of GH^dex^, with Trp362 in the side wall conformation in both apo and liganded states.

The entrance to the GH^dex^ binding cavity is stabilised by surface-exposed aromatic hydrophobic residues likely to attract glycans. Aromatic residues including Trp and Tyr are highly enriched in carbohydrate binding sites, with glycan CH groups interacting with protein p electrons (CH-p-stacking) (Kiessling and Diehl, 2021). Phenylalanine also p-stacks with glycans, but there is no significant enrichment of this residue in glycan binding sites in the Uniprot database (Hudson et al., 2015). GH^dex^ aromatics around the dextran ligand include Trp260, Tyr221, and Tyr437, all of which are ∼5 Å from the IMO3. All these residues are on solvent-exposed loops in the catalytic domain of the protein, except for Tyr221, which is on the third β-strand of the (β/α)_8_-barrel domain (β3 in Fig. 2B). There is little structural change once dextran is bound, with both apo GH^dex^ WT and E360A matching liganded GH^dex^ E360A with Cα RMSDs of ∼0.4 Å. The most notable difference is Asp171, which in the dextran-bound structure bends towards subsite −2 and is likely an important stabilising residue, forming multiple hydrogen bonds to the glycan (Fig. 2C and Fig. S5). Asp171 and His254, which hydrogen bond to subsite glucoses −2 and − 3 respectively in GH^dex^, are equivalent to Asp185 and His268 in *Tp*^dex^, which form similar bonds. Asp171 and Tyr221 are highly likely to be interacting residues, based on their hydrogen bonds with subsite −2 of IMO3 (Fig. 2C). Asp171 is on the loop after β-strand 2, and Tyr221 is on β-strand 3. Tyr437, which points to the glycosidic bond between subsites −3 and − 4, is on loop between β-strand 8 and α-helix 8 of the catalytic domain.

### 2.3 Structure determination of SGBP^dex^

The original construct designed for SGBP^dex^ did not crystallise despite extensive screening. Therefore, a truncated construct was designed to remove the N-terminal Ig-like domain (SGBP^dex^ Δ1–147, Fig. S1) which was not predicted to bind dextran based on comparison with the canonical SusF structure (Cameron et al., 2012). The truncation was based on Alphafold2 (Jumper et al., 2021) models of SGBP^dex^ and SusF, which has a homologous domain architecture to SGBP^dex^, with an N-terminal Ig-like domain followed by three Ig-like domains in tandem that each contain one glycan binding site (Cameron et al., 2012). These domains were denoted carbohydrate-binding modules (CBM) (Cameron et al., 2012), as they are ∼100 residue β-sheet-rich domains that bound starch without enzymatic activity, although they do not belong to a known CBM family. We have also used this terminology for SGBP^dex^ due to its similar fold and role in PUL48; denoted CBMα (residues 154–263), CBMβ (residues 264–378) and CBMγ (residues 379–504) SGBP^dex^ Δ1–147, containing all three putative CBMs, was successfully crystallised and the structure is highly similar to the Alphafold2 model, albeit with a different orientation between CBMα and CBMβ (PDB ID: 9SJI, Fig. S6). Crystal soaking and co-crystallisation experiments aiming to bind dextran ligands to SGBP^dex^ Δ1–147 were unsuccessful. For SusF, the CBMγ provides much of the starch-binding activity (Cameron et al., 2012), and mutagenesis of predicted tryptophan residues in SGBP^dex^ CBMγ showed this may also be the case in the dextran-binding protein (Table S2). A *cis* peptide bond identified in the crystal structure may be important for conformational change in SGBP^dex^ (Fig. S7 and Supplementary Discussion).

### 2.4 Structure determination of SusD^dex^ in the absence and presence of substrate

**Fig. 3:**
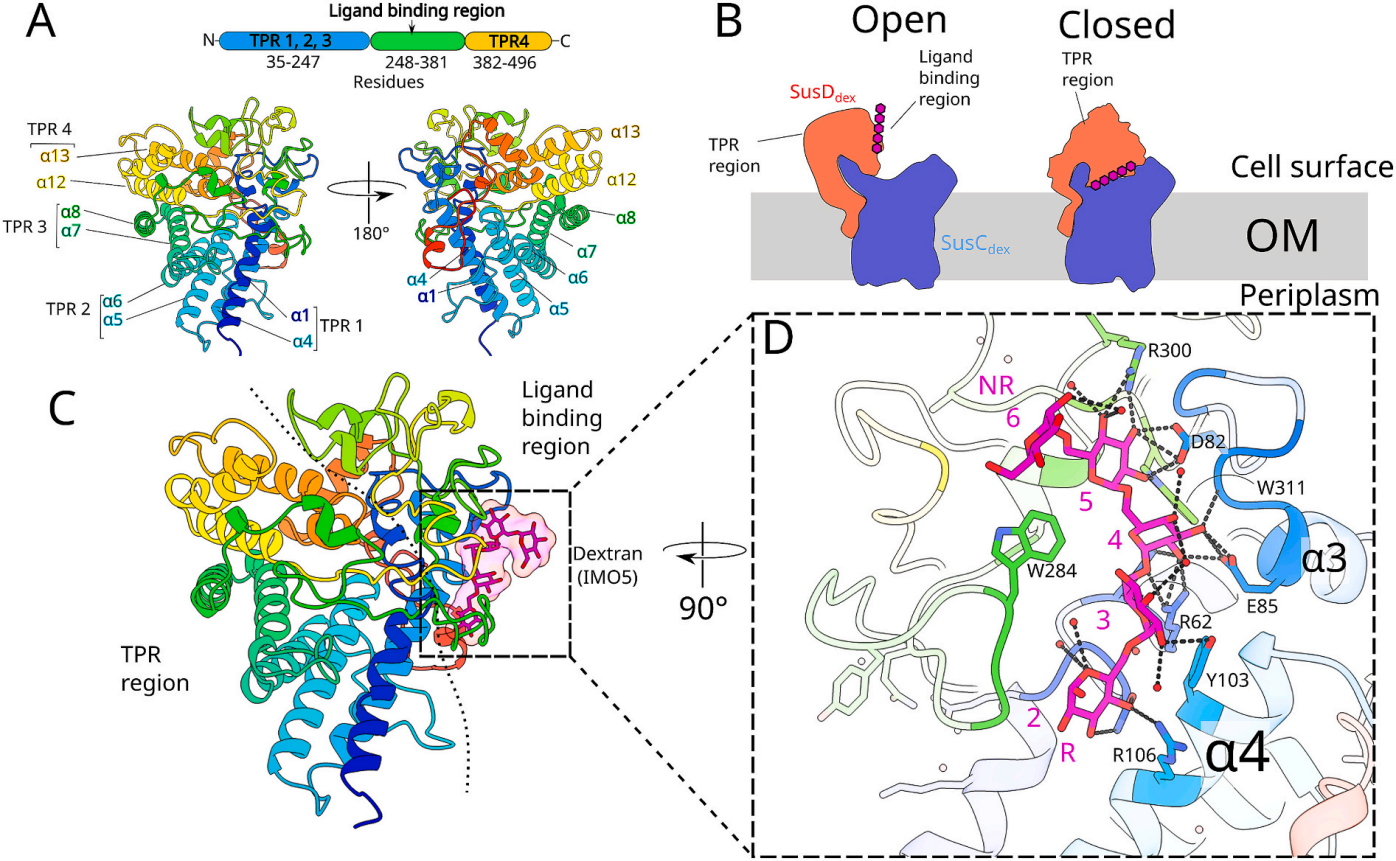
The crystal structure of apo SusD^dex^ and soaked with dextran 1.5. A. Apo SusD^dex^ viewed from an arbitrary front and back view. α helices that constitute the four TPR pairs are labelled (α1/4, α5/6, α7/8, and α12/13). In panel B, the core utilisome components of the utilisome (SusCD^dex^) are shown in the open and closed states to illustrate the substrate-capturing role of SusD^dex^ in the OM. C, IMO5–bound SusD^dex^ with a line separating the two approximate regions of the protein; the more structured and conserved TPR region, and the less structured and conserved ligand binding region. Dextran 1.5 has a DP range between 6 and 10 residues. The five modelled residues (isomaltopentaose, IMO5) of dextran 1.5 are shown in both stick and surface representation. In D, hydrogen bonds with the IMO5 are shown as dashed lines, and IMO5 residues are numbered from the reducing end (R) to the non-reducing end (NR). The numbering corresponds to the subsites defined in the cryo-EM structures (Fig. 6). SusD^dex^ structures with and without IMO5 were deposited under PDB IDs 9SJJ and 9SJK, respectively.

SusD^dex^ was crystallised and structures were solved to resolutions of 1.65 Å (apo, 9SJK) and 1.7 Å (dextran 1.5 soaked, PDB ID: 9SJJ). As expected, the fold of SusD^dex^ was typically SusD-like, and was highly similar to the Alphafold2 model (Cα RMSD = 0.8 Å). SusD^dex^ contains four tetratricopeptide (TPR) domains which form a right-handed superhelix (Bolam and Koropatkin, 2012), which constitute the cell surface-facing side (Fig. 3A). The ligand binding region (residues 248–381), which is relatively unstructured, is sandwiched between TPR 3 and 4, and forms the periplasmic-facing side. This allows SusD^dex^ to deliver bound ligands to the SusC^dex^ after lid closure (Fig. 3B), a mechanism which has been well-established across several homologues (Glenwright et al., 2017; Gray et al., 2021; White et al., 2023; Madej et al., 2020; Tong et al., 2024). The ligand binding site with bound isomaltopentaose (IMO5) is shown in Fig. 3C-D. In the ligand binding region, α9 (293–297) and its surrounding loops provide dextran-binding aromatic residues including Trp284, Trp311 and Tyr103. The α3-containing loop and α4 of TPR1 including Asp82, Glu85 and Arg106 provide a lower surface with an extensive hydrogen bonding network to dextran hydroxyl groups. Asn57 and Arg62 from the first 3_10_ helix and Arg300 from the ligand binding region also interface with the IMO5.

Like in GH^dex^, the binding of dextran to SusD^dex^ causes little to no structural change (Cα RMSD of 0.4 Å). Residues along the groove of the binding side including Asp82, Tyr103, Arg106, Trp284, Arg300 and Trp311 have similar side chain conformations between apo and dextran-bound models. The only notable difference is Glu85, which points inwards in the liganded structure, hydrogen bonding with a glycan hydroxyl in subsite 3 (Fig. 3D). In the apo structure, the position of the Glu85 carboxylate is instead occupied by a sulphate ion from the crystallisation solution and ordered water, and the residue points away from the binding site. *Privateer* glycan validation (Agirre et al., 2015), which we believe to be reliable at this resolution, found all five glucose residues to be in the chair conformation.

For comparison between *B. theta* SusDs, surfaces (coloured by hydrophobicity) of liganded SusD^dex^, SusD^starch^ and SusD^lev^ are shown in Fig. S8. Cα RMSDs of SusD^dex^ when matched to SusD^lev^ and SusD^starch^ were 6.9 Å and 9.3 Å respectively. The SusD^dex^ dextran binding site has a solvent-exposed Trp284, below which a hydrophobic groove in the protein surface is formed by Trp292, Trp311, Phe296, Trp309 and Tyr370. These residues stem from the ligand-binding region (Fig. 3C). Across from Trp284, the N-terminal α-helix 4 (which forms TPR 1 with α1) provides a solvent-exposed Tyr103 which appears to clamp the ligand between Trp284 and Tyr103. The shape of the SusD^starch^ binding site appears to be divergent from the levan and dextran SusDs (Fig. S8). Unlike SusD^dex/lev^, there is no central protruding aromatic residue (Trp284 and Phe649 for SusD^dex^ and SusC^lev^ respectively) and the ligand projects from the protein surface, instead of running parallel like for SusD^dex/lev^. This may be related to the smaller helical pitch of starch, resulting in a more curved substrate (Koropatkin et al., 2008). The groove of the binding site is more pronounced, with aromatic residues Trp85 and Tyr296 protruding from the surface, with the substrate in between. Trp96 and Trp320 form an aromatic patch between the protruding residues. The SusCD^lev^ site more closely resembles the SusD^dex^ site (Fig. S8B and D). In SusCD^lev^, the solvent-exposed Phe649 of SusC^lev^ provides the central aromatic group, equivalent to Trp284 in SusD^dex^. Below Phe649 is a groove of hydrophobic and aromatic residues of SusD^lev^ including Phe301 and Tyr395. Like Tyr103 and Trp284 in SusD^dex^, Trp85 (SusD^lev^) and Phe649 (SusC^lev^) may provide a clamp to hold levan substrate, although only Trp85 is essential for levan binding (Gray et al., 2021). Trp85 may be replicated in SusD^dex^ by Tyr103, which occupies a similar position in α4 of TPR 1 in both SusDs.

### 2.5 Cryo-EM of the inactive dextran utilisome in the presence of ligand

**Fig. 4:**
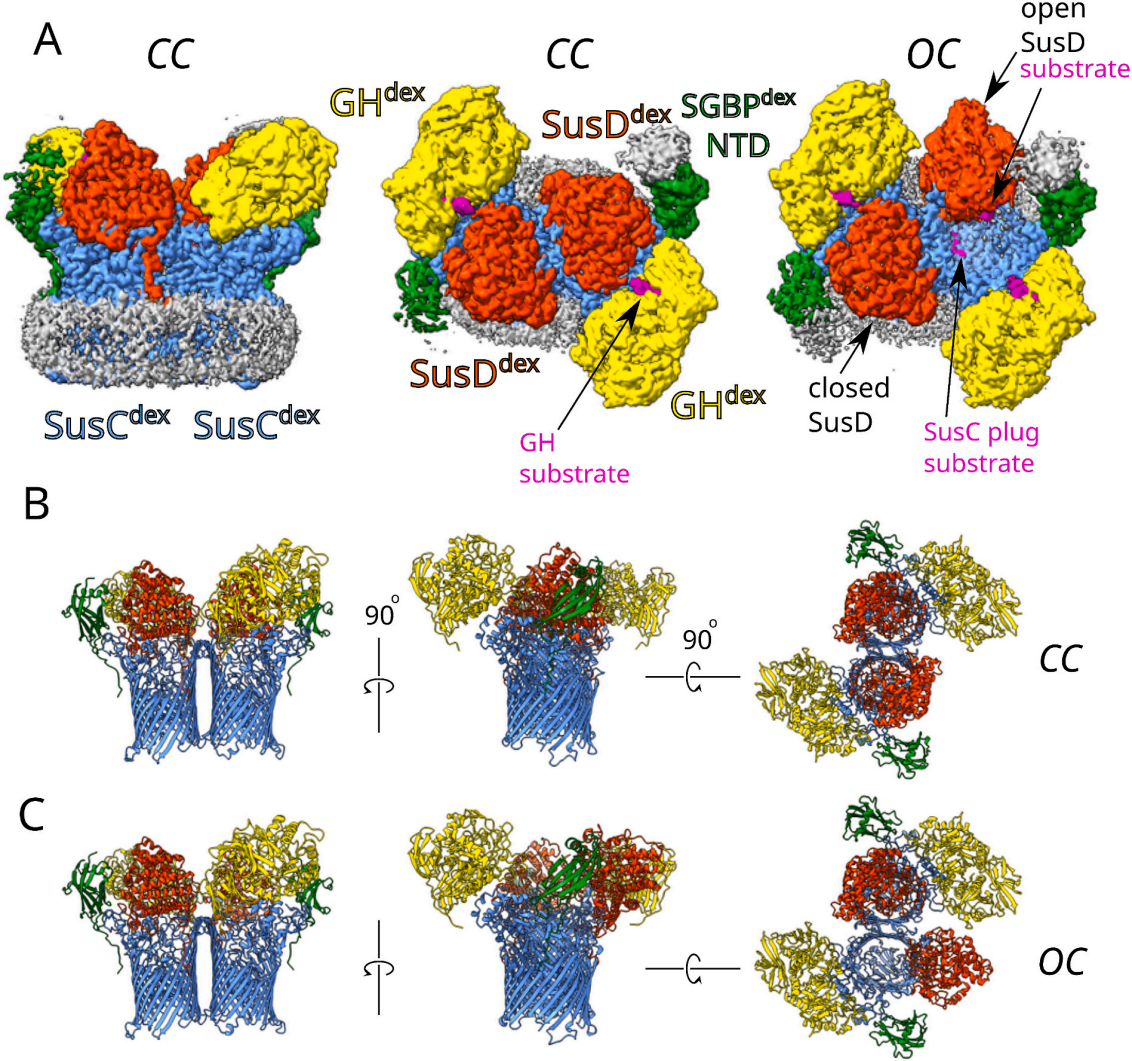
The structure of the inactive dextran utilisome bound to ligand. A. Final 3D reconstructions of the closed-closed (CC) and the open-closed (OC) states of the inactive dextran utilisome, in complex with dextran. The final CC and OC maps had nominal resolutions of 2.5 and 2.9 Å (Fig. S11C), after FSC-mask auto-tightening, respectively. The map was colour zoned according to the atomic models of the dimeric four-component complex (GH^dex^:SGBP^dex^:SusD^dex^:SusC^dex^); GH^dex^ is yellow, SGBP^dex^ is green, SusD^dex^ is orange-red and SusC^dex^ is cornflower blue. Densities with modelled isomaltooligosaccharides are magenta. Note the additional substrate densities visible in the open state. Only the N-terminal domain (NTD) of SGBP^dex^ was reconstructed at a modellable resolution, but the putative three CBM domains are visible at higher thresholds (Fig. S12). B, C Cartoons of the modelled dextran utilisome in various orientations for the CC (B, PDB ID: 9SM2) and OC (C, PDB ID: 9SJE) state. (For interpretation of the references to colour in this figure legend, the reader is referred to the web version of this article.)

We previously established that dextran and levan utilisomes are dimeric four-component complexes that are stable in the absence of substrate (White et al., 2023). To investigate how substrates bind to and potentially affect the dextran utilisome, we first generated a *B. theta* strain with the GH^dex⁎^ double mutation D297A/E360A and a His6 tag on the C-terminus of SusD^dex^ to allow the purification of the catalytically inactive utilisome. Several approaches were tried to obtain a sample that behaved well on cryo-EM grids . The best dataset was achieved through a combination approach. DDM (*n*-dodecyl-β-D-maltoside) was used for complex extraction and was then exchanged to lauryl maltose neopentyl glycol (LMNG) during IMAC (immobilised metal affinity chromatography). Excess LMNG was removed by SEC (size exclusion chromatography) in the absence of detergent (see Fig. S9 and Materials and Methods). For vitrification, UltrAuFoil grids were treated with polyethylene glycol (PEG) using a protocol to render the gold support layer hydrophilic *(*Meyerson et al., *2014**)*, and a secondary detergent (fluoro-octyl maltoside) was added. This led to more abundant particles that were distributed throughout the grid holes. A mixture of dextran 1.5 and 3 was added at 0.5 mM each and incubated for 30 mins before vitrification. Following data processing (Fig. S10–11, Table S3), the resulting reconstructions had nominal resolutions of ∼3 Å based on gold-standard Fourier shell correlation of the masked volumes (Fig. S11), with density for all protein chains of the octameric complex (2 x SusC^dex^, 2 x SusD^dex^, 2 x GH^dex^ and 2 x SGBP^dex^). The SGBP^dex^ density remained low-resolution, likely due to the flexibility of the protein, and only the N-terminal domain (NTD) could be modelled (Fig. 4A).

### 2.6 The dextran utilisome remains dynamic in the presence of substrate

Previous work on the active dextran utilisome in the absence of substrate found only classes with open SusD lids after 3D classification (White et al., 2023). In the inactive dextran utilisome plus substrate reported here, ∼60% of particles had closed SusD^dex^ lids (“closed-closed”, CC, 9SM2), while ∼40% were observed with one SusD open (“open-closed”, OC, 9SJE) (see Fig. 4 and Fig. S11A). This differed from the inactive levan utilisome where only closed states were observed upon substrate addition (White et al., 2023). Additionally, SGBP^lev^ was tethered to the rest of the utilisome by fructose oligosaccharides (FOS) of DP 15–25, which enabled its model building. By contrast, dextrans between 1 and 20 kDa did not have the same effect on SGBP^dex^ across several grids and datasets, suggesting a subtly different role for SGBP^dex^.

**Fig. 5:**
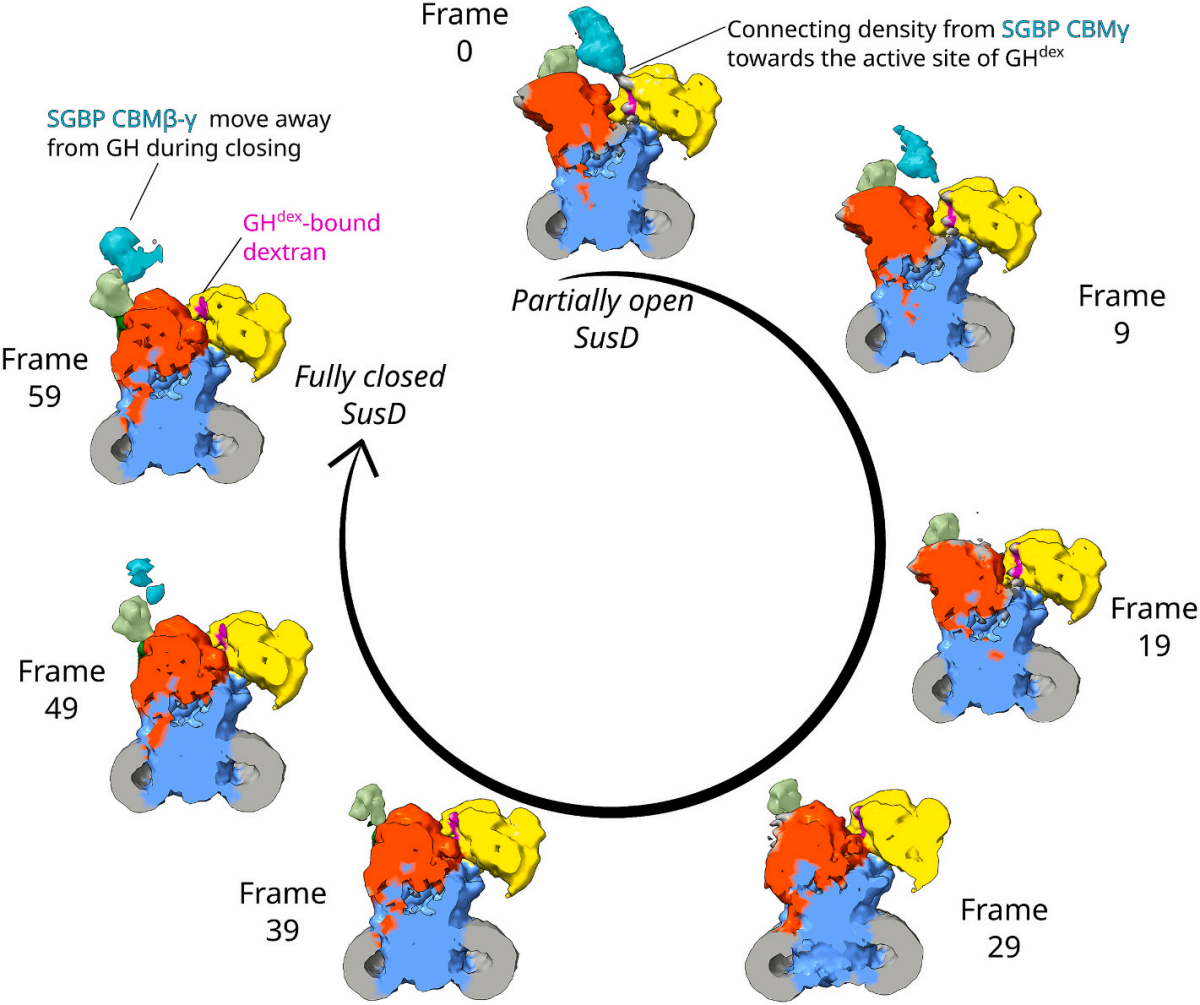
Conformational cycling in the dextran utilisome. Seven frames of the dextran utilisome transition from a partly open SusD^dex^ state (frame 0) to the closed state (frame 59) are shown, solved using *CryoSPARC* 3D variability analysis (Punjani and Fleet, 2021). The maps have been slabbed so that one copy of the protein tetramer (SusCD^dex^, GH^dex^, SGBP^dex^) is shown from the direction of the hidden second copy. To colour the map by subunit, the CC dextran utilisome model was docked into the map of each frame as two models. The OC model was not used for docking into the open frames (e.g. frame 0), as it had SusD^dex^ forming a larger opening than the states solved by 3D variability. The first model included GH^dex^, SusC^dex^ and the SGBP^dex^ NTD, while the second model docked just SusD^dex^ to account for its variable positioning between different frames. To aid in the visualisation of the unmodelled SGBP^dex^ CBMs, these were coloured by image processing software; CBMα in light green and CBMβ-γ in cyan. IMO8 and IMO4 in the SusCD^dex^ cavity were deleted and only the GH^dex^ ligand was kept due to its relatively static position throughout the motion. Density attributed to CBMβ-γ is observed transiently between frames 0–9 and 49–59. In frames 0–9, the CBMγ CTD appears to contact the GH^dex^ binding site, while in frames 49–59, CBMβ-γ was resolved on top of the closed SusD^dex^. This figure is also shown as a 20-frame transition in Movie S1. (For interpretation of the references to colour in this figure legend, the reader is referred to the web version of this article.)

The dynamics within the dextran utilisome were visualised via 3D variability analysis (Punjani and Fleet, 2021), showing the closing of SusD^dex^ (Fig. 5 and Movie S1). At the beginning of the movie (Fig. 5, Frame 0), the C-terminal domain (CTD) of SGBP^dex^ contacts the active site of GH^dex^, which may be how SGBP^dex^ supplies dextran to GH^dex^ for hydrolysis. As SusD^dex^ hinges closed, SGBP^dex^ becomes poorly resolved, but at the end of the movie the SGBP^dex^ CTD can be seen above the closed SusD^dex^ (Frame 59), translating to an approximate 40 Å shift in position of the C-terminus of the protein. To estimate the positions of SGBP^dex^ in low contour density, Alphafold-predicted domains CBMα and CBMβ-γ were docked independently in the CC and OC maps (Fig. S12). Docking into the OC map showed that the density is weakest at CBMβ, indicating that this domain is the most mobile (Fig. S12C). *Cis/trans* isomerisation of a *cis*-peptide in CBMβ, Ala306-Asp307 (Fig. S7), may help drive movement of SGBP^dex^ CBM domains upon dextran binding (see Supplementary Discussion).

### 2.7 Two extracellular hinge loops of SusC^dex^ are involved in the closing of SusD^dex^

The open and closed dextran utilisome cryo-EM models were analysed, revealing an opening of 62°, with a small shift of 2 Å (Fig. S13A). This was a relatively large opening compared to the open and closed models from the core levan utilisome ([SusCD]_2_), which only had a measured rotation angle of 23° (1 Å shift). A state of the apo levan utilisome with a larger opening was previously observed, but it was not modelled (Gray et al., 2021; White et al., 2023). The fully-open SusCD^dex^ opening is similar to open states of both ButCD and RagAB (from *Bacteroides fragilis* and *Porphyromonas gingivalis*, respectively (Madej et al., 2020; Tong et al., 2024)) based on visual inspection (Fig. S14).

In the dextran utilisome, extracellular loops 7 and 8 of SusC^dex^ act as a mobile platform for SusD^dex^, similar to the levan utilisome (White et al., 2023). Here, to emphasise their importance, SusC^dex^ loops 7 and 8 are termed “extracellular hinge loops” (L7, EHL1: 627–667 and L8, EHL2: 697–722) (Fig. S13). Movement of EHL1 and EHL2 (Fig. S13B-D) and polar interactions with SusD^dex^ (Fig. S15) may be implicated in stabilising the closure of substrate-loaded SusD^dex^. PDBePISA (Krissinel and Henrick, 2007) analysis was performed, with SusC-D^dex^ interfaces and polar interactions in the open and closed states included in the supplementary information (Spreadsheet S1). EHL1 and EHL2 make up 48% and 64% of SusC^dex^ polar interactions with closed and open SusD^dex^, respectively. SusC^lev^ EHL1 is longer than the SusC^dex^ EHL1 by approximately 20 residues. In the closed state, these residues project towards captured ligand, providing a binding surface with SusC^lev^ Phe649 at the tip of the loop (Fig. S8D) (White et al., 2023). Other important loops are loop 1 (253–303) and loop 9 (759–843) which act as binding platforms for GH^dex^ and SGBP^dex^, respectively. There is almost no conformational change in loops 1 and 9 between the open and closed models, suggesting that they are static.

Between the open and closed SusC^dex^, the tip of EHL1 shifts 21.7 Å inwards during lid closure (based on Cα positions of Gly647). Thr708, at the tip of EHL2, undergoes a smaller shift of 10.6 Å (Fig. S13C-D). Based on the loop structures in both states, EHL1 moves as a rigid body akin to a lever, while EHL2 undergoes a rotation towards the SusC^dex^ lumen stemming from the 2-stranded β-sheet at the base of the loop. The phenyl ring of EHL2 Phe718, at the top end of the β-sheet, is shifted 7 Å towards EHL1 after lid closure (based on the phenyl ring *p*‑carbon). Another consequence of the rotation is a shift of SusC^dex^ Asp713 Cα of 6.5 Å inwards towards SusD^dex^ Arg106 in the closed state, forming two salt bridges (Fig. S13E). Arg106 itself moves ∼16 Å inwards during closure, indicating these interchain salt bridges may stabilise the closed state during the transport cycle.

EHL1 forms 14 and 26 close contacts (Van der Waals overlap > −0.01 Å) with SusD^dex^ in the open and closed states, respectively. A contact in the open state to SusD^dex^ Pro166 is not maintained during closure, although nearby Phe164 contacts EHL1 in both states. Most of the additional contacts in the closed state come from α1 of SusD^dex^ (residues 39–54). A hydrophobic motif (Tyr644-Pro645-Phe646) at the tip of EHL1 contacts SusD^dex^ α1, particularly Gln54, and loop residues at the beginning of the unstructured ligand binding region (Gln274, Gln289). SusC^dex^ Tyr644 and SusD^dex^ Tyr50 appear to form an aromatic stack which is maintained during closure. After closure, residues on the loop between TPR 2 and 3 contact EHL1, including Arg208 and Asn204. By contrast, EHL2 makes 4 and 8 contacts to SusD^dex^ in its open and closed states, respectively. In the open state, three of the four contacts are to SusD^dex^ Phe164, which are broken when SusD^dex^ closes, where Phe164 contacts the closed state EHL1 instead. This suggests that SusD^dex^ Phe164 is an important residue for the hinging movement. After EHL2-Phe164 contacts are lost during closure, two tryptophans in α4 of SusD^dex^ TPR1, Trp105 and Trp109, form an interface with EHL2.

In the closed conformation, there is one close contact between SusD^dex^ Arg348 and GH^dex^ Asp364, which is not observed in the open conformation. Closer inspection revealed that an electropositive patch of SusD^dex^ (Arg348, Arg347) is near an electronegative patch of GH^dex^ (Glu365, Asp364, Asp367), which may provide rigidity in the closed complex. These residues do not form salt bridges in the model, but the inter-residue distances appear to be in the proper range for interaction (3–4 Å). The levan utilisome does not share this feature. The modelled portion of SGBP^dex^ (N-terminal domain; 21–154) forms no contact with SusD^dex^ or GH^dex^ in either the open or closed state.

### 2.8 Substrate binding sites in the dextran utilisome

**Fig. 6:**
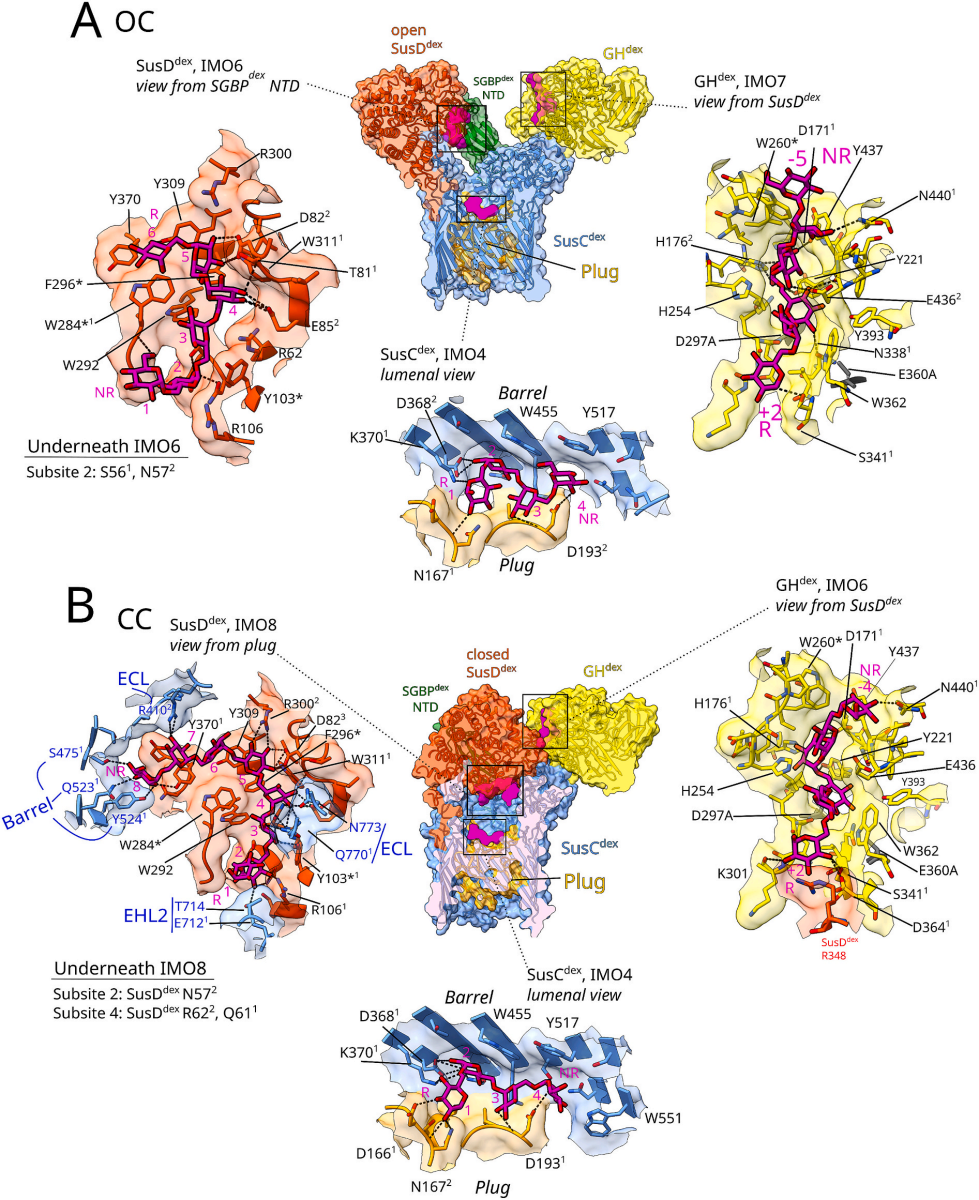
Dextran binding sites in GH^dex^ (yellow), SusD^dex^ (orange-red) and SusC^dex^ (blue) in the open (A) and closed (B) utilisome. Colour scheme is consistent with previous figures, with the models are clipped to view the lumen of SusC^dex^. Overviews of the utilisomes are shown in the centre. Dextran oligosaccharides are shown in magenta, with protein-glycan hydrogen bonds shown as black dotted lines. “IMOx” are isomalto-oligosaccharides where x denotes the number of α-ᴅ-glucose residues. Superscript numbers on the residues denote how many atoms are hydrogen bonding to dextran. For both models, each binding site is magnified and displayed at the most informative orientation. Protein residues up to 5 Å from the ligands are displayed in stick representation, with aromatic and other notable residues are labelled. The subsites are numbered by glucose residue, with non-reducing (NR) and reducing ends (R) labelled. For GH^dex^, these follow the convention of increasingly negative numbering from the cleavage site towards the non-reducing end (Davies et al., 1997). SusC^dex^ residues are grouped and labelled based on what part of SusC they originate from; EHL2 and ECL refer to extracellular hinging loop 2 and extracellular loops, respectively. Barrel refers to residues on the beta-strands, and plug refers to the SusC^dex^ plug domain (residues 129–232). (For interpretation of the references to colour in this figure legend, the reader is referred to the web version of this article.)

The binding sites of GH^dex^, SusD^dex^ and SusC^dex^ in the open and closed utilisome models are shown in Fig. 6 and Movie S2 (see also Table S6), with an overview in Fig. S16. For GH^dex^, differences between the open and closed states are very minor with a Cα RMSD of 0.5 Å. GH^dex^ has numerous aromatic glycan binding residues that define the IMO7 (OC) and the IMO6 (CC) binding pocket (Fig. 6A-B). From the “top” (OC, subsite −5) to the “bottom” (subsite +2) these are; Trp260, Tyr437, His254, His176, Phe438, Tyr221, Tyr396, Tyr393, and Trp362. When viewing GH^dex^ from the direction of SusD^dex^, three residues (Trp362, Tyr393 and Tyr396) form the right wall of the binding site. In the closed state, these three residues are shifted slightly inwards, away from SusD^dex^ that comes into close contact with GH^dex^ after hinging. Trp260, at the top of the binding site, is important for dextran binding given that its substitution by Ala led to a 62-fold decrease in *K*_d_ for dextran 1.5 (DP = 6–10) (Table S2). A substitution of Trp362 to Ala could not be expressed, indicating that it may be required for protein folding. At these resolutions (> 2.0 Å), it is not possible to confidently say which is the correct orientation of the glycan chain. The reducing end was chosen to point downwards, towards SusD^dex^ in both models (Fig. 6A, B). In the closed GH^dex^ model, the IMO6 conformation is similar to the crystal structure of *Tp*^dex^ (Suzuki et al., 2016) (Fig. S2D), with the non-reducing end (subsite −4) pointing away from SusC^dex^ (Fig. 6B), and from the catalytic residues at the bottom of the binding site.

Closed state SusD^dex^ has the largest bound dextran ligand observed (IMO8), whereas in the open state only part of the binding site was stably occupied (IMO6, subsites 1–6). Since the local map resolution of the open SusD^dex^ was much lower than its equivalent in the closed state (∼3–5 Å vs ∼2 Å, Fig. S17), it is not surprising that less dextran density was observed. The lower resolution is likely due to flexibility of the open SusD^dex^, and the fact that the OC map had many fewer particles than the CC map (∼21,000 vs. 116,000). By contrast, the closed SusD^dex^ state is stabilised by several interactions with SusC^dex^, minimising flexibility (Fig. S15).

Like in the crystal structure of liganded SusD^dex^, dextran forms a planar semi-circle around the central upper aromatic binding residue Trp284. Based on ITC, Trp284 and Phe294 were essential for binding of dextran 1.5, while Tyr103 was beneficial but not necessary (Table S2). Closed SusD^dex^ forms 12 contacts with its IMO8 ligand (Van der Waals overlap > −0.01 Å), primarily via hydrogen-bonding residues, including Asp82, which has 3 contacts with subsite 5. Near the reducing end, subsite 2 is also closely associated with SusD^dex^, with 2 contacts with Asn57, and 1 with Arg106. Moving along the glycan, subsite 3 contacts Tyr103, while subsite 4 contacts Gly83. Tyr370 also makes a contact within its phenolic ring to subsite 7. Interestingly, compared to closed SusD, open SusD^dex^ forms more contacts (17 vs. 12) with its smaller IMO6 ligand. Contacts with hydrogen bonding residues Asp82, Asn57 and Arg106 are maintained, but additional aromatic-ligand interactions are present, particularly at subsite 6, which has 3 contacts to Trp284 and 2 to Tyr307. While the overall ligand poses between the crystal structure and both cryo-EM states of SusD^dex^ are highly similar, the open SusD^dex^ ligand differs at its terminal subsite 6, which bends inwards towards Trp284. This suggests that Trp284 provides CH-π-stacking interactions to dextran oligosaccharides during transport that may be broken after lid closure, contributing to ligand release.

In the closed state, SusC^dex^ has several residues that hydrogen bond with IMO8. These SusC^dex^-glycan hydrogen bonds (Fig. 6B, in blue) and SusCD^dex^ salt bridge interactions (Fig. S15) likely help stabilise the closed state. Starting at the non-reducing end, several residues at the top of adjacent β-barrel strands bond to subsite 8 (Ser475, Gln523, Tyr524, Fig. 6B). Extracellular loop (ECL) residues Arg410 and Gln770 bond to subsites 7 and 3, respectively. EHL2 Glu712 hydrogen bonds to subsite 2, while Thr714 of EHL2 forms a close contact to subsite 1, which is the only SusC^dex^ residue to do so, possibly indicating subsite 1 as a favoured endpoint for substrate loading (Fig. 6B, EHL2). Additionally, Asp713 is sandwiched between these residues, and forms a protein-protein salt bridge with the nearby SusD^dex^ Arg106 (Fig. S13E). This suggests the EHL2 ^712^Glu-Asp-Thr^714^ motif may have a multi-functional role in both ligand stabilisation and lid closure.

When the contour of the CC map is lowered (Fig. S18), the closed SusD^dex^ IMO8 density extends further another two glucose residues towards the binding site of the SusC^dex^ plug domain, but this section of density was not modelled. SusC^dex^ Tyr477 is proximal to the unmodelled density at IMO8 (Fig. S18, model only), which like Tyr542, faces the interior of the barrel. At still lower map contour (contour 0.017–0.010), the IMO density extends from both ends of IMO8 (A and B) towards the SusC^dex^ plug site, while IMO4 (C and D) extends in the opposite direction, suggesting some complexes contain longer dextran oligosaccharides that span both the SusD^dex^ and SusC^dex^ binding sites simultaneously. Based on this weak density, we estimate a dextran size limit of around DP 17 (∼3 kDa) could be present in the SusCD^dex^ cavity, in rough agreement with the levan utilisome (White et al., 2023).

In the SusC^dex^ plug binding site, isomaltotetraose (IMO4) was modelled in both states, although the density was weaker for open SusC^dex^. In both models, the main glycan binding residue appears to be SusC^dex^ Trp455, from the luminal face of β-strand 7. SusC^dex^ conformations around both open and closed plug sites are almost identical, indicating no major changes occur at the plug between open and closed states. Subsites 1 and 2 form the majority of close contacts to Lys370, Gly425 and Trp455 of the β-barrel, as well as to Asp166 and Asn167 of the plug domain. Residues involved in hydrogen bonding include β-barrel residues Asp368 and Lys370, as well as Asp166/193 and Asn167 from the plug domain (Fig. 6A and B, orange surface). IMO4 conformations are highly similar for subsites 1 and 2. In the open transporter, the IMO4 subsites 3 and 4 are bent inwards, with the subsite 4 CH-π-stacking with Tyr517, while in the closed state, IMO4 subsite 4 is wedged between Tyr517 and Trp551. Although the side chain of Trp551 is not displayed in Fig. 6A for the open state (due to the different IMO4 conformation and the 5 Å radius requirement from the ligand for residue display), it is in an almost identical conformation to that in the closed state (Fig. 6B).

### 2.9 The dextran utilisome, like the levan utilisome, has an aromatic lock

**Fig. 7:**
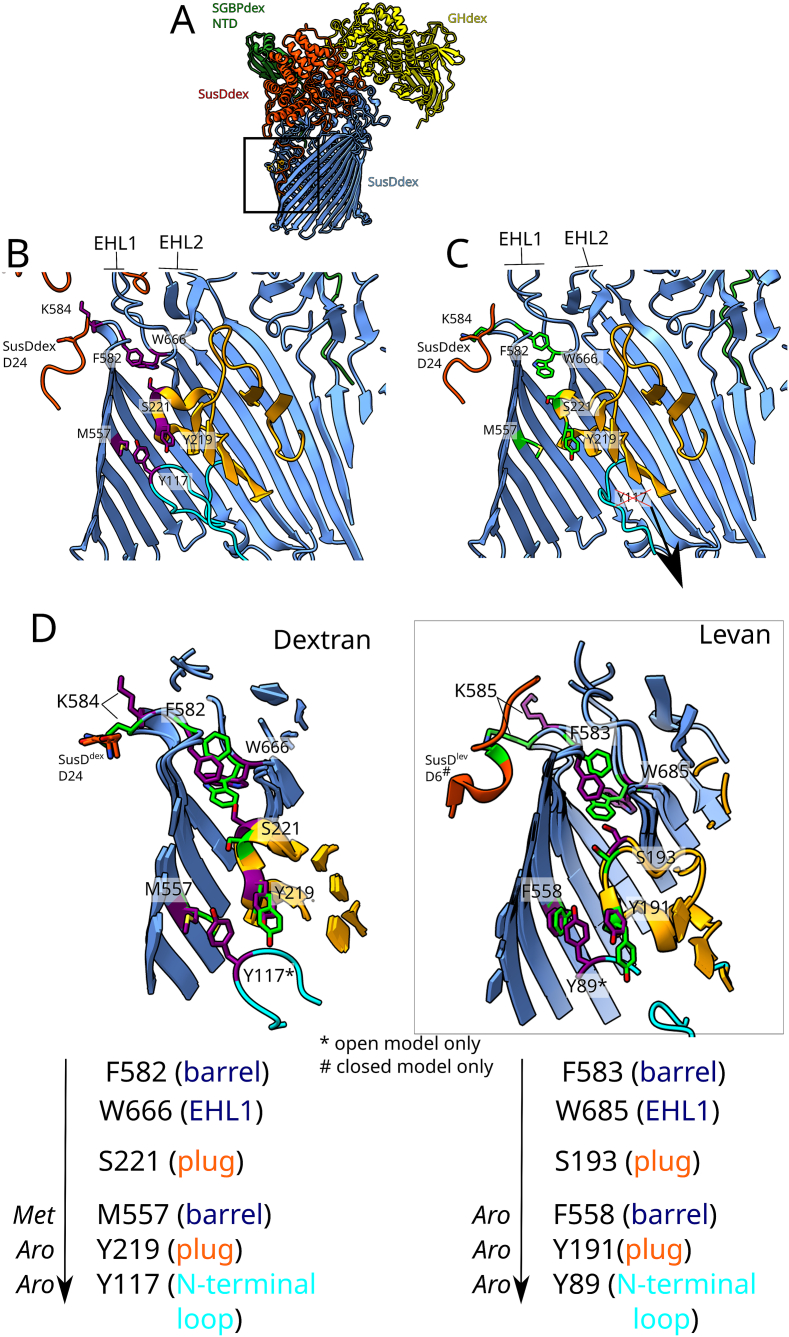
The “aromatic lock” that facilitates release of the N-terminal TonB box of SusC^dex^. The colour scheme is consistent with previous figures (SusC^dex^ blue, SusD^dex^ orange-red, SGBP^dex^ green and GH^dex^ yellow). The plug domain of SusC^dex^ is in orange while the N-terminal TonB box-containing loop is in cyan. A. The relevant area of the utilisome is highlighted. B—C. The open (B) and closed (C) models are shown in detail, with residues that undergo shifts between the models displayed. These include a methionine and two aromatics at the inner face of the barrel (Met557, Phe582, Trp666), the plug domain (Tyr219, Ser221), and the TonB box containing N-terminal loop (Tyr117, resolved only in open SusC^dex^). These residues are coloured purple (open state) or in lime green (closed state). Tyr117, which interacts with Met557 and Tyr219 in the open state, forms the aromatic lock, sequestering the TonB box inside the SusC^dex^ lumen. After closure of SusD^dex^, these interactions are broken, releasing the N-terminal loop into the periplasm for TonB to access the TonB box. D. Overlay of the open and closed states in the dextran and levan systems. Levan models include the active transporter, where no SusD^lev^ was modelled, but they are in the open state (PDB ID: 8A9Y) and the inactive, closed SusD^lev^ model (PDB ID: 8AA0). In the bottom panel, an arrow shows the direction of the allosteric signal from the top of SusC^dex^ (Phe582) to the N-terminal loop (Tyr117). The location of each shifted residue is in brackets. (For interpretation of the references to colour in this figure legend, the reader is referred to the web version of this article.)

The “aromatic lock” of SusC-type TBDTs was first described in the levan utilisome (White et al., 2023). Comparison of the substrate-bound and apo levan utilisome structures showed that in the apo utilisome, Tyr89, Tyr191 and Phe558 form a triple aromatic stack that physically links the TonB box (via Tyr89, which is next to TonB box residues Asp82-Gly88), the plug domain (via Tyr191) and the inner wall of the β-barrel (via Phe558). Upon substrate binding, Trp685 shifts further into the barrel, moving Phe583 up and Ser193 down. This movement causes the Tyr191-containing-loop to move further towards the periplasm, breaking its interaction with Tyr89 and releasing the TonB box-containing loop into the periplasm. Consequently, in the substrate-bound structure this loop is not modelled as it is too flexible to be resolved, but the last resolved residue in both structures (Arg93) undergoes a 35 Å shift. This displacement/disordering of the SusC N-terminus is likely necessary for signaling substrate binding across the OM, allowing the TonB protein to access the TonB box sequence in the periplasmic space (Fig. 7).

The aromatic lock is conserved between the levan and dextran utilisomes. It is important to note that residue numberings are shifted between these structures, as the levan utilisome polypeptides were numbered from the first mature residue whereas the dextran utilisome polypeptides were numbered including their N-terminal signal sequences (i.e. the SusC^lev^ numbering is offset by −25). The SusC^dex^ β-barrel-loop residues Phe582 and Trp666 are homologous to SusC^lev^ Phe583 and Trp685. SusC^lev^ Phe558, which is internally facing from a β-strand, is absent in SusC^dex^, with a methionine in its place (Met557). This means the aromatic stack in the dextran utilisome is a “double” (Tyr219 and Tyr117) instead of a “triple” stack described for the levan utilisome (White et al., 2023). However, methionine residues are a common bridge between two aromatic residues in an Aro-Met-Aro fashion (Weber and Warren, 2019), indicating that Met557 likely has a similar role to SusC^lev^ Phe558, as it occupies the same position on the barrel wall (Fig. 7D).

Allosteric substrate signaling appears to begin at SusC^dex^ Trp666, at the end of EHL1, where it flips towards the plug in the closed state (Fig. 7). This has two consequences. Firstly, Phe582 is nudged upwards by Trp666, slightly reordering the β-barrel-loop residues 579–587. This causes Lys584 to point towards SusD^dex^ Asp24 on the N-terminal linker of the the SusD^dex^ lid, forming an additional salt bridge (to Asp24-Arg587, which is present in both open and closed states, Fig. S15) which may stabilise lid closure (Fig. 7B-C). A similar trend is observed in the position of equivalent lysine Lys585 in the active (open) and substrate bound (closed) levan utilisome structures, with Lys585 forming a salt bridge with SusD^lev^ Asp6 in the closed state (Fig. 7D). Secondly, the downwards movement of the Trp666 indole ring causes Ser221 to flip away, carrying the signal to the plug domain. The nearby plug residues Ile218 and Tyr219 clash with Tyr117 of the N-terminal loop, breaking the Tyr-Met-Tyr stacking interaction between Tyr117, Met557 and Tyr221. Ile218 is shown in detail in a closer view of the interfacing N-terminal loop, plug domain and barrel (Fig. S19). The equivalent residue in SusC^lev^, Ile190, does not shift with its neighbouring Tyr191, but instead slightly rotates. The resulting N-terminal disordering and movement allows the TonB box to be accessed by TonB in the periplasmic space: the first residue resolved in closed SusC^dex^, Thr119, is shifted ∼16 Å slightly down and away to the opposite end of the barrel lumen compared to its position in the open state (Fig. S20). The first resolved residue of open SusC^dex^ (Leu109) occupies a similar position to where Thr119 is in the closed model.

### 2.10 Isothermal titration calorimetry shows varying affinities of the SLPs for dextrans

**Fig. 8:**
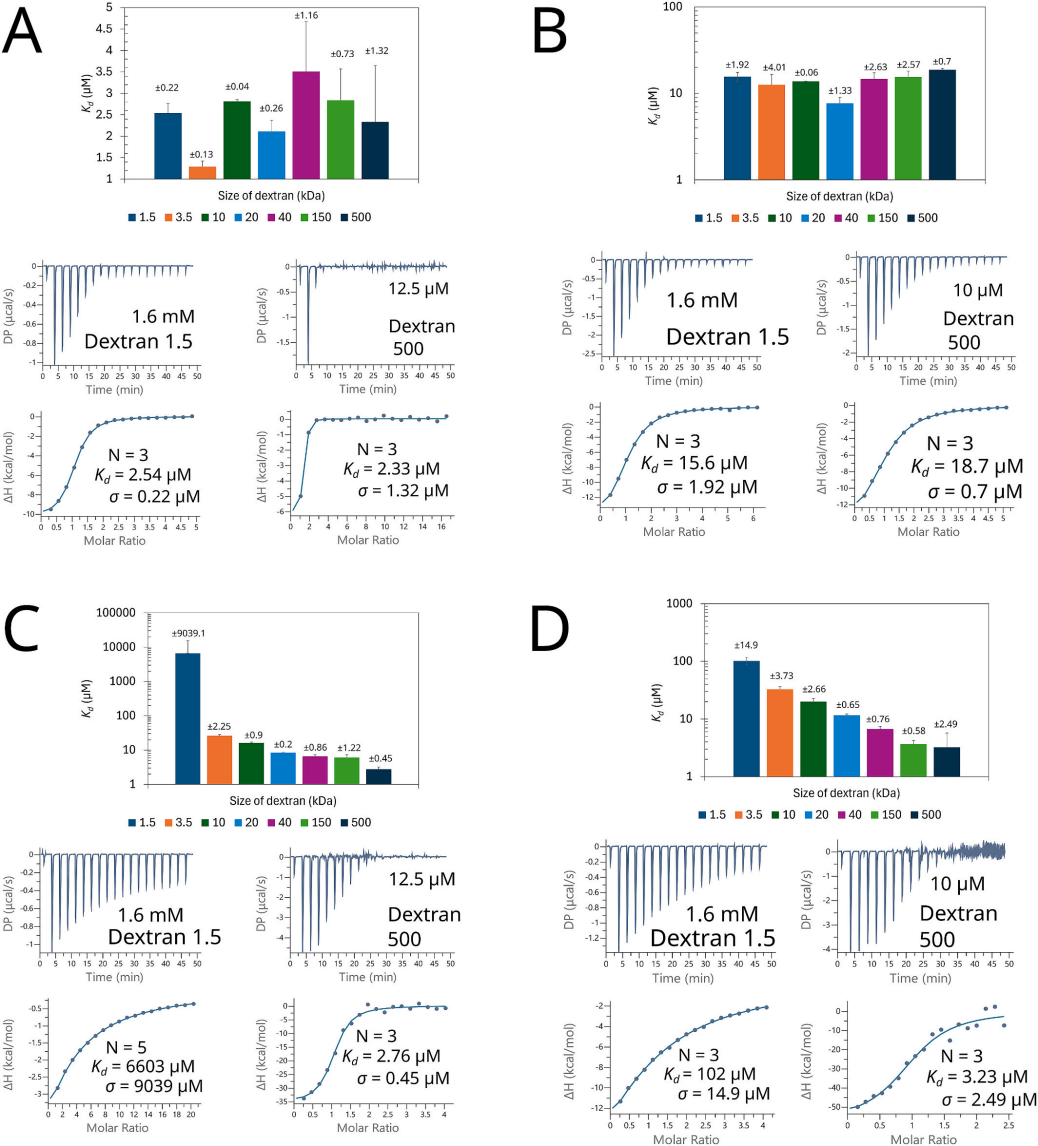
Dextran substrate affinities of GH^dex^ E360A (A), SusD^dex^ (B), SGBP^dex^ (C) and SGBP^dex^ Δ1–147 (D) against dextrans from 1.5 to 500 kDa. Derived *K*_d_ values from Table S4, which fixed the ITC *n* value to 1, are plotted on logarithmic scales (SusD^dex^, SGBP^dex^ and SGBP^dex^ Δ1–147) or a linear scale (GH^dex^ E360A) against dextran size. Note that the Y axes differ in scale between the different proteins. Bars are colour-coded by dextran size. Error bars represent the standard deviation (*σ*), calculated using the *K*_*d*_ derived from the replicates. The standard deviation is also included above each bar, expressed in μM. Example titrations for each protein are shown below for dextran 1.5 and dextran 500. N On the thermograms, N refers to the number of replicates. More example titrations are shown in Spreadsheet S2.

Dextran-binding activity was quantified by titrating dextrans from 1.5 to 500 kDa into purified SLPs (Table S4). SusD^dex^, SGBP^dex^, SGBP^dex^ Δ1–147, and two catalytically inactive mutants of GH^dex^ (E360A and D297A/E360A) were used. GH^dex^ had the highest binding affinity for dextran (1–4 μM), independent of dextran size. SusD^dex^ affinities were lower, in the 8–20 μM range. Both GH^dex^ and SusD^dex^ showed little difference between the size of dextran and *K*_d_. By contrast, dextran binding affinities of SGBP^dex^ and SGBP^dex^ Δ1–147 depended strongly on ligand size (Fig. 8).

SGBP^dex^ had a very low affinity (6 mM) for dextran 1.5 with high variability between measurements due to the poor suitability of ITC to measure low-affinity interactions. Affinities increased with the size of dextran; when SGBP^dex^ and SGBP^dex^ Δ1–147 were titrated with dextran 500, *K*_*d*_ values comparable to GH^dex^ were obtained (∼3 μM), indicating that SGBP^dex^ preferentially binds large dextrans that have not yet been processed by GH^dex^.

To infer the number of binding sites of these dextran-binding proteins, two of the smallest dextrans available from Pharmacosmos (technical grade dextran 1.5 and 3.5) were compared in a separate ITC data analysis using the same raw data. The *n* value was allowed to vary to estimate dextran-to-protein stoichiometry (Table S5). We hypothesised that dextran 1.5 was small enough (DP = 6–10) that it could not bind more than one binding site simultaneously. These results were then compared to dextran 3.5 (DP = 16–22), and *n* values were compared. This suggested SusD^dex^ had one binding site for dextran 1.5 (derived *n* = 1.01) and half this for dextran 3.5 (*n* = 0.49), indicating that glycans of DP 6–10 are typical substrates for SusD^dex^. This agreed with predictions that SusD^dex^ would contain one binding site based on homology and previous structural data (Glenwright et al., 2017; White et al., 2023; Bolam and Koropatkin, 2012).

For GH^dex^ E360A, titrations of dextran 1.5 (*n* = 2.12) indicated that two dextran molecules were bound per protein. Conversely, dextran 3.5 gave an average value of 0.94, indicating that this dextran (DP ∼20) may be large enough to span two putative binding sites in GH^dex^. In the cryo-EM structure of the substrate-loaded levan utilisome, as well as the crystal structure of GH^lev^, two sites were observed on GH^lev^: one forming the active site and another on top of the catalytic domain (White et al., 2023). These sites did not bind the same levan ligand simultaneously, but this may be possible with longer ligands in solution. However, unlike GH^lev^, the crystal and cryo-EM structures of GH^dex^ did not suggest two distinct binding sites. SusG from the starch PUL also contains two binding sites, but this is provided by a distinct CBM domain (Fig. S4) (Koropatkin and Smith, 2010). These GHs belong to distinct families from GH^dex^; GH13 and GH32 respectively. The second closest structural homologue in the PDB, GH66 family member *Tp*^dex^, had only one binding site (Suzuki et al., 2016).

SGBP^dex^ is homologous to SusF of the starch PUL, which was shown to have three binding sites for glycans, with one on each CBM (Cameron et al., 2012). By contrast, the structure of SGBP^lev^ is divergent with only one binding site (White et al., 2023). *n* value analysis was unreliable for SGBP^dex^ and SGBP^dex^ Δ 1–147, likely due to the presence of several binding sites and its low affinity for small dextrans (Table S5). While we captured 1:1 stoichiometry in the cryo-EM structure for SusD^dex^ and GH^dex^ and their dextran substrates, the SGBP^dex^ CBM domains were poorly resolved. We believe this may be partially due to inherent flexibility of SGBP^dex^, but also due to different dextran binding modalities across the three putative binding sites. Furthermore, the binding site of CBMβ may have a role in dextran-generated conformational changes of CBMβ-γ (Supplementary Discussion). It is worth noting that the Trp → Ala SGBP^dex^ CBMγ variants produced in the current study had relatively high dextran affinity (∼5-fold reduction) compared to the Trp → Ala GH^dex^ (∼62-fold reduction) or SusD^dex^ variants (no binding) (Table S2 and Spreadsheet S2), perhaps suggesting that multiple CBM are involved in dextran binding. However, there are other factors that contribute to this, requiring further investigation.

In Δ*BT3088 B. theta* grown on dextran 200 (DP ∼1111), lag time was significantly increased, while growth rate and maximum OD600 were significantly reduced when compared to cells grown on dextran 35 (DP ∼195) (Wong et al., 2024). Δ*BT3088* cells also exhibited impaired cell surface localisation of dextrans greater than 40 kDa, based on a fluorescent dextran study (Wong et al., 2024). While we acknowledge that the conditions of SLP ITC assays are far removed from the utilisome in situ, this data and the ITC support the idea that the SGBP^dex^ has a unique role in the binding and utilisation of higher molecular weight dextrans. We suggest that this may arise from cooperativity of the three CBM domains (α, β, and γ), allowing larger dextrans to span multiple binding sites simultaneously. In the 3DVA analysis (Fig. 5, Movie S1), we see the potential for binding site cooperativity, as the GH^dex^ docked substrate appears to interface with CBMγ of SGBP^dex^. Additionally, we previously saw a novel state in the levan utilisome where both copies of SGBP^lev^ bind the same substrate, with one protein reaching over to its symmetry mate (White et al., 2023). However, we did not observe anything of this nature in the cryo-EM data for SGBP^dex^.

Further study is needed to elucidate the role of each CBM domain, likely using methods orthogonal to cryo-EM and ITC. The testing of recombinant CBM domain variants with ITC, analogous to the deletion work on SusF (Cameron et al., 2012) could be employed to help understand the contribution of each domain, as could genomic deletion of CBMs combined with growth assays. In the context of the *B. theta* OM, one could imagine clustered utilisomes consisting of several SGBP^dex^ which all bind the same high-molecular-weight dextran substrate. Investigating this would require new approaches, such as cryo-electron tomography of *B. theta* membranes or fluorescence studies in live cells.

## 3 Discussion

**Fig. 9:**
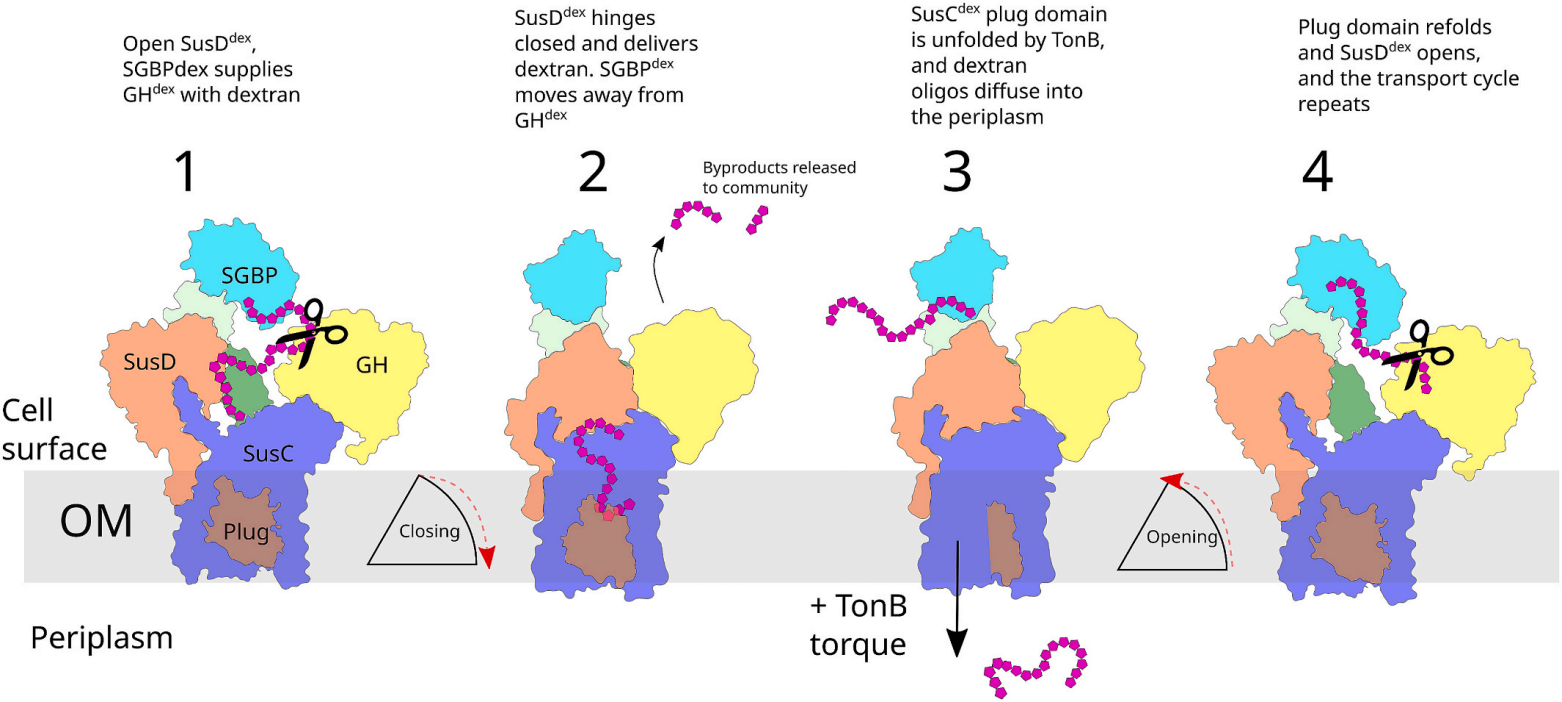
A schematic representation of the dextran utilisome transport cycle. Four-component utilisomes, rather than the full octameric complexes, are shown for clarity. Side views based on the OC (1 and 4) and CC (2 and 3) models are shown in the OM plane. GH^dex^ is yellow, SusD^dex^ is orange red and SusC^dex^ is cornflower blue. For SGBP^dex^, the dark green portion is the modelled NTD, while the pale green (CBMα) and cyan (CBMβ-γ) sections show the docked positions of the CBM domains. Magenta pentagons are dextran oligosaccharides. (For interpretation of the references to colour in this figure legend, the reader is referred to the web version of this article.)

Fig. 9 shows the proposed transport cycle of the dextran utilisome, which was based on both the cryo-EM-derived OC and CC models, in combination with the 3D variability data. In step 1, CBMγ of SGBP^dex^ contacts the GH^dex^ active site (Fig. 5, Fig. S12, Movie S1), supplying dextran to be hydrolysed endolytically (Kim et al., 2012a). The products then bind to the open SusD^dex^ lid. The observation of bound ligand to open SusD is interesting and suggests that ligand binding does not immediately result in lid closure akin to a venus flytrap mechanism (at least in vitro). Some dextran is likely released into the environment post-GH^dex^ hydrolysis, facilitating the cross-feeding of other microbiota members (Wong et al., 2023). When the SusD^dex^ lid closes, the transporter lumen is loaded (step 2). An open question is whether lid closure occurs spontaneously (due to natural dynamics of the lid) or due to a yet unknown trigger. Previous single-channel physiology experiments on plug-less SusCD^lev^ in the absence of substrate showed very noisy current traces with large amplitudes (∼1.5 nS), indicative of rapid (< ms) opening and closing of the SusD lid (Glenwright et al., 2017). In addition, cryo-EM structures of apo SusCD^lev^ lacking the additional SLP components show open and closed SusD lids (Gray et al., 2021). A prerequisite for the spontaneous dynamics model is that closure of an empty SusD lid should not lead to TonB box exposure since that could result in a non-productive transport cycle. However, complete levan utilisomes purified under mild conditions showed exclusively open SusD lids without substrate and closed lids with substrate (White et al., 2023), suggesting a substrate binding-driven lid closure in vivo. Indeed, closed SusCD complexes *without* bound ligand have not yet been observed, and we therefore favour a substrate-driven model of “stable” lid closure. This model might explain why TBDT lids evolved in the first place: if it takes a long time for the interaction with TonB to occur, a stably closed lid would prevent dissociation of the ligand back into the extracellular space, as would happen with a regular, “lidless” TBDT. Another consideration is that it is not clear what influence, if any, the native OM environment (likely dominated by negatively charged lipo-oligosaccharides on the cell surface (Jacobson et al., 2018)) has on lid dynamics.

During lid closure, SGBP^dex^ moves away from GH^dex^ (Fig. 5, Movie S1), and stabilising salt bridges are formed between SusC^dex^ and SusD^dex^ (Fig. S15, Supplementary information). An allosteric signal cascade travels down SusC^dex^ to disrupt the aromatic lock, causing a conformational change of the SusC^dex^ N-terminus that exposes the TonB box for interaction with the inner membrane TonB-ExbBD complex (Braun et al., 2023; Zinke et al., 2024) (step 3; TonB-ExbBD complex not shown). Rotary proton pumping by ExbBD causes conformational differences in TonB, generating a pulling force which then partly unfolds the SusC^dex^ plug domain, creating a transient pore (Silale and van den Berg, 2023). IMO products are then able to diffuse into the periplasm. After dissociation of the TonB box-TonB interaction, the plug refolds and SusD^dex^ opens for a new transport cycle (step 4).

## 4 Materials and methods

### 4.1 Cloning of SusD^dex^, SGBP^dex^ and GH^dex^ for *E. coli* expression

The following protocol was used to generate soluble constructs for the PUL48 surface-exposed lipoproteins *BT3087*, *BT3088* and *BT3089.* To facilitate crystallisation, primer pairs were designed for each construct to remove the N-terminal signal sequence and an additional ten amino acids of the mature lipoprotein, including the cysteine that would be tri-acylated in *B. theta*. Genomic sequences of *Bacteroides thetaiotaomicron* VPI-5482 for primer design were obtained from KEGG (https://www.genome.jp/), and primers were synthesised by Eurofins genomics. N-terminal signal sequences were predicted by supplying the amino acid sequence to SignalP 5.0 (Almagro Armenteros et al., 2019). The constructs contained residues 26–592, 31–504, and 29–496 for *BT3087*/GH^dex^, *BT3088*/SGBP^dex^, and *BT3089*/SusD^dex^ respectively. *BT3088 (Δ1–147)* contained residues 148–504, corresponding to a deletion of the N-terminal domain. Sequences of *BT3087*, *BT3088* and *BT3089* were amplified from purified *B. theta* genomic DNA with primers containing *Nco*I and *Xho*I restriction sites, restricted and ligated into pET28b, giving the constructs a C-terminal His6-tag for purification. BT3088 (Δ1–147) was N-terminally His-tagged with a forward primer containing the codon (CAC) x 6, after the C-terminally His-tagged variant failed to bind to the column during IMAC purification. Chemically competent *E. coli* TOP10 cells were then transformed using heat shock. Positive *E. coli* TOP10 clones were identified by colony PCR of individually picked colonies that were grown on an antibiotic selection plate after ligation. These constructs were designed to be overexpressed in the *E. coli* cytoplasm by isopropyl ß-D-1-thiogalactopyranoside (IPTG) induction of the *T7lac* promoter.

### 4.2 Expression of SusD^dex^, SGBP^dex^ and GH^dex^

LB media (Granulated Lennox broth, Melford Laboratories) was dissolved in deionised water at 20 g/L and autoclaved. Freshly transformed BL21 (*DE3*) *E. coli* on LB-agar plates with 50 μg/mL kanamycin (Melford) were used to inoculate precultures which were propagated overnight at 37 °C and 1/100th of the culture volume was typically used as inoculum (e.g., 10 mL per 1 L final culture). Cells transformed with the pET28b vector were selected using 50 μg/mL kanamycin. After growth to exponential phase (OD600 = 0.4–0.8), IPTG (Melford) was added to final concentrations between 0.2 mM and 1 mM to induce protein expression. Different expression conditions after induction were tested, such as 37 °C at 180 rpm for 2–4 h or 16–20 °C, 150 rpm for 19–22 h until acceptable conditions were found for each construct. Cultures were then harvested for 25 min at 4 °C either by centrifugation in a Beckman J6-HC (integrated rotor) at 4200 rpm, or in a JLA-8.1000 rotor (Beckman Coulter) at 5500 rpm (8456 x *g*) in an Avanti J-26 XP centrifuge (Beckman Coulter).

### 4.3 Purification of SusD^dex^, SGBP^dex^ and GH^dex^

*E. coli* cell pellets with overexpressed SLP were suspended in Tris Buffered Saline (TBS; 20 mM Tris-HCl, 300 mM NaCl, pH 8.0) by glass dounce homogeniser. DNase I (Roche) was added at 200 μg per litre of culture (overnight culture) or 40 μg/L (3 h culture). The resuspended cells were lysed at 20 kpsi with a TS Series 0.75 kW cell disruptor (Constant Systems), followed by centrifugation of the lysate at ∼43,000 g for 30 min to separate the soluble proteins. Cell pellets and lysate were kept on ice (or in centrifuges cooled to 4 °C) during initial processing, with IMAC and SEC performed at room temperature.

An IMAC column of with ∼3 mL volume of Sepharose resin (Chelating Sepharose Fast Flow, Cytiva) was prepared with 0.1 M nickel chloride, washed with MilliQ water, and then equilibrated with TBS (+30 mM imidazole). The supernatant was then loaded by gravity-flow onto the IMAC column. The column was then washed with TBS (+30 mM imidazole), and SLPs were eluted with 5–10 mL of TBS (+200 mM imidazole). Proteins were buffer exchanged and concentrated using Amicon Ultra 15 centrifugal filters with a molecular weight cut off (MWCO) of 30 kDa (Merck Millipore).

Subsequently, SEC was carried out on a Superose 6 Increase 10/300 GL column (10 mM HEPES, 100 mM NaCl, pH 7.5). The use of Superose resin was necessary as the PUL48 SLP proteins all interact with the frequently used, dextran-based Superdex resin, causing very late elution volumes. Protein aliquots were stored at 4 °C for brief periods before rapid flash freezing in liquid nitrogen before use for X-ray crystallography or ITC.

### 4.4 Cloning of *BT3087* (GH^dex^) catalytic mutants and dextran binding mutants in *BT3087*, *BT3088* and *BT3089*

Putative catalytic residues in *BT3087* were identified from the literature (Kim et al., 2012a; Kim et al., 2012b). The Q5 site-directed mutagenesis kit (New England Biolabs) was used to introduce the D297A and E360A mutations in the previously cloned pET-28 *BT3087* construct using a novel primer pair designed with New England Biolab's online tool (https://nebasechanger.neb.com/). This protocol involved whole-vector PCR with primers harbouring the gene modifications. After the single mutants were confirmed by Sanger sequencing (Eurofins Genomics), a double mutant (D297A, E360A) was produced using a confirmed single mutant as template DNA. The same method was used to create dextran-binding mutants of *BT3087* (GH^dex^), *BT3088* (SGBP^dex^) and *BT3089* (SusD^dex^).

### 4.5 Dextran digestion assay

Dextran 500 (Biosynth, range of 450–550 kDa, average 500 kDa) was dissolved at 5 mg/mL in 10 mM HEPES-NaOH, 100 mM NaCl, pH 7.5 with 66.6 μM of either wild-type or GH^dex^ E360A (single catalytic mutant) that had been purified from BL21 (*DE3*) *E. coli.* Each sample was incubated at 37 °C / 400 rpm in an Eppendorf Thermomixer and aliquots from each reaction were taken at various time points to assess the progress of the reactions. Aliquots were immediately boiled in a heat block for 15 min to inactivate the enzymes, followed by a 5-min centrifugation step at ∼17,000 x*g*. Aliquots were subsequently frozen at −20 °C for later thin-layer chromatography analysis. The time points were at 0 (*i.e.* before GH^dex^ was added), 1, 5, 10, 30, 60, 120, 240 min, and a final overnight time point (21.5 h after initiation).

### 4.6 Thin-layer chromatography

Foil-backed silica TLC sheets (Silica gel 60 F254) were cut to size and a line was drawn 1 cm from the bottom edge in pencil. Samples were loaded 1 cm apart from each other along the line by pipetting 2 μL at a time followed by drying with a hairdryer. For oligosaccharide samples, 6 μL was loaded per spot. The first and last lanes were reserved for a size marker consisting of 3 μL of 1 mM glucose, maltose and isomaltotriose, corresponding to dextran digestion products with a DP of 1, 2 and 3 respectively. The loaded sheets were then placed in a grease-sealed glass tank with ∼0.5 cm of butanol, acetic acid and water mixture at a ratio of 2:1:1. After approximately 2.25 h, the running buffer was <1 cm from the top of the sheets, and they were removed from the tank before being carefully dried with a hairdryer. To stain the migrated sugars, the sheet was briefly submerged in a developer solution (sulphuric acid, ethanol and water; 3:70:20 *v*/v with 1% orcinol) for a few seconds, and then carefully dried again. Stained sheets were then taken to a heated cabinet (120 °C) for a few minutes until the bands developed sufficiently.

### 4.7 Isothermal titration calorimetry analysis of purified dextran utilisome lipoproteins

Titrations were performed for GH^dex^ E360A, GH^dex^ D297A/E360A, SGBP^dex^, SGBP^dex^ Δ1–147, and SusD^dex^ on a Microcal PEAQ-ITC (Malvern Panalytical). Dextran solutions were used as the injected titrant, while the proteins were the analytes inside the ITC cell. The concentration of protein was typically 25–50 μM, while dextran varied from 1.6 mM (dextran 1.5) to 10–12.5 μM (dextran 500). Dextrans 1.5 and 3.5 were sourced from Pharmacosmos (technical grade), with larger dextrans sourced from Biosynth. The dextran concentrations were optimised experimentally to obtain as many injections covering the inflection point of the ITC curve as possible. The standard procedure started with 10 min of equilibration at 25 °C at a reference power of 10 μcal/s set at 750 rpm of stirring speed. The first injection of 0.4 μL of the titrant (ignored in the analysis) was followed by 18 injections of 2 μL. The initial delay was 60 s, and injections lasted 4 s and were spaced with 150 s pauses. As initial heats of injection were very large, the contribution of dilution heats was assumed to be minimal, and the “fitted offset” option was used to correct for baseline heat contributions.

Since dextran is highly soluble in water, and the commercially available dextrans come as powders, dialysis was not performed. Dextran solutions were made up to particular molar concentrations based on their average molecular weight. However, commercial dextrans are only purified to a certain degree and there is some uncertainty in the exact polymeric contents. For example, technical grade dextran 1.5 (Pharmacosmos) has a molecular weight range of 1200–1800. Dextrans were dissolved in the same buffer as the protein (10 mM HEPES-NaOH, 100 mM NaCl, pH 7.5) and briefly heated to ∼50 °C until fully dissolved, and then aspirated with a pipette or vortexed as required.

Three different analyses were used on the Microcal PEAQ-ITC software. Firstly, to calculate the overall affinities presented in Fig. 8, the “one set of sites” model was used with the number of binding sites (*n*) was fixed to 1 (Table S4), allowing the concentration of the dextran ligand to vary from the calculated concentration (*e.g.* 1.6 mM of dextran 1.5) to fit the data. Protein concentration was fixed at 30 μM for GH^dex^ E360A and D297A/E360A, 25 or 50 μM for SGBP^dex^, 41.3 μM for SGBP^dex^ Δ1–147, and 50 μM for SusD^dex^. The appropriate dextran concentration was determined through trial and error. Three replicates were used for each titration, except SGBP^dex^ with dextran 1.5 which used four titrations with 25 μM protein and one with 50 μM.

Secondly, for stoichiometry analysis (Table S5) the same model and data was used but *n* was allowed to vary instead of the concentration of dextran ligand. Protein concentration was fixed at 30 μM for GH^dex^ E360A, 25 μM for SGBP^dex^, 41.3 μM for SGBP^dex^ Δ1–147, and 50 μM for SusD^dex^. Three replicates were used for each titration, except SGBP^dex^ with dextran 1.5, which used four.

Thirdly, binding mutations were also assessed (Table S2, *n* value = 1). Protein concentration was fixed at 36 μM for GH^dex^ D297A/E360A W260A, 30 μM for GH^dex^ D297A/E360A (“WT”), 25 μM for all SGBP^dex^ constructs, and 57 μM for SusD^dex^ Y103A or 50 μM for WT, SusD^dex^ W284A, and SusD^dex^ F296A. To control the comparisons, the dextran ligand concentration was fixed per-comparison (*e.g.* the same for SusD^dex^ WT and its variants). To calculate *K*_d_ fold-change of a variant compared to its wild-type, the same wild-type data were refitted with the calculated dextran concentration and protein concentration both fixed, so they could be compared to titrations of binding variants where the same parameters were also fixed. This meant the wild-type *K*_*d*_ listed in Table S2 were slightly different to the overall affinity analysis in Table S4. When binding was detected, mutant titrations were repeated to obtain triplicate data. GH^dex^ D297A/E360A W260A was collected only in duplicate due to insufficient protein availability. Non-binding mutants were also collected only in duplicate.

### 4.8 Crystallisation of GH^dex^, SGBP^dex^, SusD^dex^

After size-exclusion chromatography, purified proteins were concentrated to 10–50 mg/mL using 15 mL Amicon centrifugal filters (MWCO = 30 kDa) (Merck Millipore). Proteins were screened by sitting drop vapour diffusion crystallisation using the PACT, Structure, Morpheus I and JCSG+ screens (Molecular Dimensions), the Index screen (Hampton Research), and if the target crystallised in ammonium sulphate conditions, the AmSO4 screen was also used for hit optimisation (QIAGEN). For each of the 96 conditions of the screens, the Mosquito (SPT Labtech) deposited mixtures of protein and crystallisation solutions; 100:100 nL (1:1) and 200:100 nL (2:1) respectively. MRC 96-Well 2-Drop Crystallisation plates (Molecular Dimensions) were used and sealed with ClearVue sheets (Molecular Dimensions). To optimise confirmed protein crystals, the Dragonfly (SPT Labtech) was used to mix a series of conditions based on the contents of the original crystallisation condition, typically varying the amount of precipitant and/or salt by 20% above and below the original condition. Crystallisation plates were kept at 20 °C and inspected regularly with an optical stereomicroscope for crystal formation. A polarising lens was sometimes used to check for birefringence in protein crystals.

All crystals were grown at 20 °C. Wild-type GH^dex^ was crystallised in a MES-containing (0.1 M, pH 6.0) ammonium sulphate condition (1.6–2.4 M). Apo GH^dex^ E360A was crystallised in 2 M ammonium sulphate, 0.2 M potassium sodium tartrate, 0.1 M sodium citrate, pH 5.6. The IMO3-bound GH^dex^ E360A was co-crystallised with dextran 1.5 (5 mM) and grown in 1.6 M ammonium sulphate, 0.5 M lithium chloride. The final apo SusD^dex^ crystal was grown in 0.8 M ammonium sulphate, 0.1 M citric acid, pH 5. An initial crystal was also grown in 50% *w*/*v* PEG 400, 0.2 M lithium sulphate, 0.1 M sodium acetate pH 4.5, yielding a marginally lower resolution structure. The IMO5-bound SusD^dex^ structure was obtained by soaking crystals grown in the PEG 400 condition with 50 mM dextran 1.5, which caused the crystals to crack into pieces. Fragments were harvested and yielded the dextran-bound structure. Initial SGBP^dex^ Δ1–147 crystals were grown in 25% w/v PEG 4000, 0.2 M ammonium sulphate, 0.1 M sodium acetate, pH 4.6. Optimised SGBP^dex^ Δ1–147 crystals were grown based on Morpheus H8 (Precipitant mix 4 [MPD, PEG 1000, and PEG 3350] 35.5% *v*/v, 200 mM amino acid stock [0.2 M alanine, glutamate, lysine, serine and glycine], 100 mM system 2 buffer [0.1 M HEPES sodium salt, 0.1 M MOPS, pH 7.5]). Crystals were harvested with appropriately sized, 20 μM thick nylon loops (Hampton Research), and cryo-protected in a 4:1 mixture of reservoir solution and either 100% PEG 400 or MPD, 6 M NaCl, or 3.5 M ammonium sulphate matching the crystallisation condition. Crystals from the Morpheus screen did not need to be cryo-protected. Crystals were then flash frozen in liquid nitrogen before being stored in canes in a cryo dewar.

### 4.9 X-ray crystallography data processing

X-ray crystallography data was processed using the CCP4cloud software suite (Krissinel et al., 2022). A typical workflow consisted of the following. Spot-finding, indexing and integration was performed using *DIALS (*Winter et al., *2022**)*. After this, space group was estimated with *POINTLESS (*Evans*, 2011**),* and scaling, merging and high-resolution cut-off determination with *AIMLESS* (Evans and Murshudov, 2013). Datasets were truncated based on outer-shell statistics for CC_half_ and mean(I) / σ(I), where ideally the former was greater than 0.5 and the latter greater than 1.0, with greater importance given to CC_half_. Asymmetric unit content determination via Matthews coefficient was performed by the CCP4 implementation (Winn et al., 2011), and molecular replacement was carried out with *phaser* and *molrep (*McCoy et al., *2007**;* Vagin and Teplyakov, 2010*)*, followed by refinement using *refmac5* and *refmacat* (Murshudov et al., 1997; Yamashita et al., 2023). *CCP4build (*Bond and Cowtan, 2022*)* was used for early model building and refinement where large modifications were required. Computer-assisted model building was performed in *Coot* (Emsley and Cowtan, 2004) and cycles of *refmac5* refinement and model-building proceeded until the model could not be improved, primarily judged by R_free_ values and *MolProbity (*Grosse-Kunstleve et al., *2002**)* scores after refinement. Modelled waters (represented as red spheres) were found and checked primarily with *Coot*. *CheckMyBlob* (Kowiel et al., 2019) was used to model unknown ligands into the difference map. A full list of data collection statistics, derived from the final *Refmac* refinements for each crystal structure can be found in Table S1. For GH^dex^ with IMO3, a Polder map of the IMO3 substrate was generated using *Phenix* (Liebschner et al., 2017).

### 4.10 Tagging of the catalytically inactive dextran utilisome in *B. theta*

*B. theta* VPI 5482 strains were created using a previously described counter-selection protocol (Gray et al., 2021; White et al., 2023). The pExchange-tdk (pEX) vector (Koropatkin et al., 2008) was used to introduce mutant genetic sequences from *E. coli* S17 λ *pir* to the genome of *B. theta (tdk-)* through conjugation and homologous recombination. Cells that contained pExchange-tdk were then selected, resulting in either wild-type or mutant recombinants, which were confirmed by both colony PCR and Sanger sequencing of the amplified region of interest. As the *BT3087 D297A/E360A* region of interest was previously cloned by site-directed mutagenesis of the *BT3087* pET28b construct, a pEX-compatible insert (with *Sal*I/*Xba*I restriction sites) was amplified using the construct as a template, and then ligated directly into pEX. Once the *BT3087 D297A/E360A B. theta* strain was confirmed, the genomic DNA of the previously generated (White et al., 2023). *BT3089*-His_6_ *B. theta* strain was amplified as a pEX insert, and then the counter-selection protocol was used to introduce *BT3089*-His_6_ into the *BT3087 D297A/E360A* background strain (Kim et al., 2012a). Colony PCR using a His-tag specific primer, and Sanger sequencing of the target region both confirmed the presence of the His-tag.

### 4.11 Expression and purification of the inactive dextran utilisome

Brain–heart infusion media (supplemented with 1 μg/mL haemin), was inoculated from *B. theta* glycerol stocks and precultured inside an anaerobic A35 workstation (Don Whitley Scientific) at 37 °C. Precultures were then used to inoculate defined minimal media (Gray et al., 2021), supplemented with 1 μg/mL haemin. To induce PUL48, 0.5% (*w*/*v*) dextran 3 MW 2500–4000 (Biosynth) was added to the minimal media, as opposed to the previously used (White et al., 2023) dextran T3.5 MW 3000–4000 (Pharmacosmos). Cells were grown anaerobically for 20–22 h and typically reached OD600 of 1.9. *B. theta* was grown as standing cultures, without any agitation. Cells were pelleted identically to *E. coli*. As *B. theta* pellets are loose compared to *E. coli*, the supernatant was poured from cell pellets immediately after centrifugation. Pellets were resuspended in TBS and frozen at −20 °C. Cells were homogenised with glass douncers and 40 μg/L DNase I (Roche) was added before lysis at either 20 kpsi (frozen cells that have been thawed) or 25 kpsi (freshly cultured cells) using one-pass on a TS Series 0.75kw cell disruptor cooled to 4 °C. *B. theta* lysate was then ultracentrifuged at 42,000 rpm (205,471 x *g*) for 50 min using a 45 Ti fixed angle rotor (Beckman Coulter) in an Optima XE Ultracentrifuge (Beckman Coulter). Isolation of *B. theta* membranes was carried out in cooled centrifuges (4 °C) and samples were kept on ice. The supernatant was then decanted, and membranes were stored for up to 3 days at 4 °C. The remainder of a purification was completed on the same day. For the cryo-EM sample, 4 L of cell cultures of *BT3087 D297A/E360A, BT3089-His6* were used to extract cell membranes. Membrane pellets were homogenised in TBS containing 2% DDM and incubated whilst stirring for 1 h in the cold room (∼6 °C) to solubilise membrane proteins. DNase I was added (2.2 μg/mL) to suspensions prior to incubation. Insoluble material was then pelleted by a second ultracentrifugation step, using the same parameters as for the lysate, but for 30 min. Gravity flow IMAC columns (∼3 mL column volume) were prepared as previously described (*Purification of SusD*^*dex*^*, SGBP*^*dex*^ *and GH*^*dex*^) for SLP purification but were kept in the cold room throughout use. Columns were first equilibrated in TBS (1.5% DDM), before the membrane protein supernatant was loaded slowly (∼50 mL/h) onto the column. The column with bound protein was then washed with 25 mL of TBS (0.15% DDM, 30 mM imidazole), followed by 25 mL of TBS (0.005% LMNG, 30 mM imidazole). For elution, 5 mL of TBS (0.002% LMNG, 200 mM imidazole) was pipetted onto the resin. After concentration with an Amicon Ultra centrifugal filter (MWCO = 100 kDa), room temperature SEC was performed with a Superose 6 Increase 10/300 GL column, as the use of a Superdex 200 column is precluded with dextran-binding proteins due to strong interactions. The column was run with size exclusion buffer without detergent (10 mM HEPES-NaOH, 100 mM NaCl, pH 7.5) to minimise excess LMNG in the final sample. Protein aliquots were stored at 4 °C during the purification before flash freezing in liquid nitrogen for storage, and later thawed for cryo-EM.

### 4.12 Cryo-EM sample preparation and data collection

For better particle distribution and number on cryo-EM holey grids, UltrAuFoil R 2/2 grids on 200 gold mesh were chemically modified with a self-assembled layer of PEG (Meyerson et al., 2014). The grids were first glow-discharged at 20 mA for 90 s on each side (total of 180 s), transferred into an anaerobic glovebox (< 2 ppm O_2_) and submerged in ethanol containing 5 mM hexa(ethylene glycol)mono-11-mercaptoundecyl ether (675,105, Sigma Aldrich) for ∼24 h. Just prior to use, the grids were removed from the glovebox and washed successively in three tubes of ethanol and left to air-dry. For vitrification, dextran T1.5 (Pharmacosmos) and dextran 3 (Biosynth) were each added to a final concentration of 0.5 mM in 7.5 mg/mL of inactive dextran utilisome, and equilibrated for at least 30 min at room temperature before vitrification. Fluorinated octyl maltoside (O310F, Anatrace) was also added at its critical micelle concentration (0.7 mM). 3 μL of utilisome was applied to the grid held in a Thermo Scientific Vitrobot IV plunge freezer. The chamber was kept at 4 °C with 100% relative humidity. After a 3 s blot delay, a blot force of −5 was applied for 6 s. For data collection, a remote collection was performed on the Titan Krios II at eBIC (electron Bio-Imaging Centre, Diamond Light Source). 12,408 movies were collected at a magnification of 105,000× using a Gatan K3 detector, resulting in a physical pixel size of 0.831 Å. Electron dosage was 23.1 e/Å^2^, and nominal defocus values were increments of 0.2 μm between −1.0 and − 2.0 μm. See Table S3 for details.

### 4.13 Cryo-EM data processing of the inactive dextran utilisome

*CryoSPARC (*Punjani et al., *2017**)* was used for processing cryo-EM movies (Table S3). Two processing pipelines were performed. For pipeline 1 (Fig. S10), patch motion correction and patch CTF were first performed on imported movies, followed by “Curate exposures” to remove low-quality images. Template picker (using 2D references from earlier datasets) was used for particle picking. Most particles were removed through cycles of 2D classification and picking of the best-looking classes. Ab-initio reconstruction was performed with two output classes to separate “junk” particles from “real” ones. Iterative rounds of non-uniform (NU) refinement and 2D classification were then used to remove more junk particles while increasing the particle box size up from 128 pixels, until the refinements were not Nyquist limited, based on interpretation of Fourier-shell correlation (FSC) curves. This generated a consensus reconstruction of 273,062 particles. 3D classification was then performed on this subset and found distinct classes for “closed-closed” (CC) and “open-closed” (OC) utilisomes. CC and OC particle subsets were then non-uniform refined separately (175,481 and 97,400 particles respectively). Another round of 3D classification was used for both particle stacks. This did not improve the resolution for the CC map, but the two best classes with a fully open SusD were chosen from the 3D classification for the final OC reconstruction of pipeline 1, which was used for model building. Using the consensus particle stack, 3D variability analysis (Punjani and Fleet, 2021) was performed which led to the SusD^dex^ hinging movie (see Fig. 5, Movie S1 and Table S6).

For pipeline 2 (Fig. S11), the dataset was reprocessed for compatibility with the *CryoSPARC* “reference-based motion correction” job, which can lead to improvement in map quality. Particle numbers in Table S3 refer to maps output from pipeline 2. Patch motion correction and patch CTF were applied to the movies, followed by “Curate exposures” to remove low-quality exposures. Particles were blob picked (140–160 Å), extracted into 400 pixel boxes, and then subjected to several rounds of 2D classification. Ab-initio reconstruction and homogenous refinement (1 class) were performed on the remaining 481,934 particles, followed by ab-initio (5 classes) and homogenous refinement, followed by ab-initio and heterogenous refinement (5 classes each). This yielded two noise classes, classes for OC and CC utilisomes, and one class of the core SusCD complex. The OC (∼150,000 particles) and CC (∼215,000 particles) classes were independently non-uniform (NU) refined to 3 and 2.9 Å, respectively. Reference-based motion correction and global and local CTF refinement was performed independently on OC and CC particle stacks, resulting in nominal resolutions of 2.5 and 2.3 Å for each state, respectively. To reduce the compositional and conformational heterogeneity of these consensus reconstructions, 3D classification (10 classes) was then performed with “force hard classification” on. For the CC stack, the best class with density for all eight protein units was used for final model building, with 116,104 particles producing a 2.5 Å reconstruction. The OC stack contained more conformational heterogeneity with classes containing different degrees of open SusD^dex^. Classes 1, 5 and 9 had a fully open SusD^dex^ and refined to 2.7 Å when their particles were combined, but the density for one copy of GH^dex^ was poor. The best octameric fully open class (class 1, 21,574 particles) was refined to 2.9 Å and used for final OC model building.

Unsharpened maps, mirrored into the correct hand, were used for model building. The cryo-EM structure of SusC^dex^ (PDB ID: 8AA4) and X-ray structures of GH^dex^ and SusD^dex^ reported in this study (PDB ID: 9SJG, 9SJK) were rigid body docked into the density and refined with several rounds of *Phenix (*Liebschner et al., *2019**)* real space refinement and semi-automated model building in *Coot* (Emsley and Cowtan, 2004). An Alphafold2 model of SGBP^dex^ was also docked, but only the NTD was sufficiently resolved in the map, so the CBM domains were deleted from the model. Dextran ligands were built using glucose (GLC) monomers and O6 to C1 were covalently linked with the *Coot AceDRG* plugin (Long et al., 2017). *Privateer* (Agirre et al., 2015) was used to provide glycan-specific restraints for further refinement. Finally, global and local *Isolde* (Croll, 2018) simulations were used to validate the model composition and lessen clashes and geometric outliers, and its output was supplied to *Phenix* for a final refinement.

### 4.14 Accession numbers

The OC and CC cryo-EM models of the dextran utilisome (Table S3) were deposited in the PDB under PDB IDs 9SJE and 9SM2, respectively. The corresponding cryo-EM maps were deposited in the EMDB under EMD IDs EMD-54945 and EMD-55024, respectively. Crystal structures (Table S1) were deposited in the PDB under PDB IDs 9SJG (GH^dex^ WT), 9SJH (GH^dex^ E360A, apo), 9SJF (GH^dex^ E360A, with IMO3), 9SJI (SGBP^dex^ Δ1–147), 9SJK (SusD^dex^, apo), and 9SJJ (SusD^dex^, with IMO5).

## CRediT authorship contribution statement

**Matthew Feasey:** Writing – review & editing, Writing – original draft, Visualization, Investigation. **Augustinas Silale:** Investigation. **Arnaud Baslé:** Software, Resources. **Bert van den Berg:** Writing – review & editing, Writing – original draft, Visualization, Supervision, Project administration, Investigation, Conceptualization.

## Declaration of competing interest

The authors declare that they have no known competing financial interests or personal relationships that could have appeared to influence the work reported in this paper.

## Data Availability

Data will be made available on request.
