# Supplementary material for: Structural and functional characterisation of the dextran utilisome from Bacteroides thetaiotaomicron
Source: J Struct Biol X. 2026 Jul 17;14:100153. doi: 10.1016/j.yjsbx.2026.100153 (PMC13450635; doi:10.1016/j.yjsbx.2026.100153)
Supplement: Supplementary file 1 [file mmc1.docx]

**Supplementary Information**

**Structural and functional characterisation of the dextran utilisome from *Bacteroides thetaiotaomicron***

Matthew Feasey, Augustinas Silale, Arnaud Baslé and Bert van den Berg*

^1^ Biosciences Institute, The Medical School, Newcastle University, Newcastle upon Tyne NE2 4HH, UK.

* Corresponding author: bert.van-den-berg@ncl.ac.uk

**Figure S1. Superose 6 SEC (size exclusion chromatography, Superose 6) chromatograms of GH^dex^, SGBP^dex^, and SusD^dex^ after recombinant expression in *E. coli* and IMAC (immobilised metal affinity chromatography, nickel affinity) purification.** The SGBP^dex^ Δ1-147 and GH^dex^ E360A variants used for crystallisation are also shown. Other GH^dex^ variants (D297A, and D297A, E360A double mutant) had similar elution profiles to the E360A variant, which were notably different to the wild-type, perhaps due to an oligomerisation unique to the wild-type. Pooled fractions are marked with “p” on the chromatograms. SDS-PAGE (sodium dodecyl sulphate–polyacrylamide gel electrophoresis) gels of IMAC samples (either column load (Lo), flowthrough (FT), wash (W), or final elutions (Elu)) and selected SEC fractions of each peak were loaded. Arrows indicate the final protein in the gel images. SEC samples were labelled corresponding to peak letters. Note that for SEC, Superose 6 columns had to be used since all the dextran binding proteins bind to (dextran-based) Superdex SEC resins.

**Figure S2. Assessing the dextran degradation activity of GH^dex^ wild-type and E360A. A.** thin layer chromatography plate showing glycan reaction products from WT GH^dex^ (left) or GH^dex^ E360A (right) digestion of dextran 500. Stained saccharides are separated by size, where smaller saccharides migrate the furthest upwards. On the x-axis are time points of the dextran digestion assay and an overnight sample taken after ~19 hours (O/N). The loaded marker (M) was 3 µl of a mixture of glucose, maltose and isomaltotriose (IMO3) at 1 mM each. (B) Marker control to check for appropriate amount of sugar to load and to confirm the three bands that were not as clearly seen on the main TLC plate. An enrichment of a larger glycan was noted over the time course, which may result from the secondary activity of GH^dex^ to catalyse the formation of cycloisomaltosaccharides (CI) [1].

**Figure S3. Comparison of dextranase structures. A.** Orientation panel showing a top-down view of the dextran utilisome complex. **B-D.** Surface and atomic model views of the co-crystal structure of GH^dex^ E360A (left) with dextran 1.5 (DP = 6-10) and the same views of thermophilic dextranase *Tp*^dex^ D312G (right) complexed with isomaltohexaose (PDB ID: 5AXH). B and C show the enzymatic site from the SusD^dex^ in the complex (in orange-red), while panel D slices through the GH^dex^ (in yellow) to present a side view. In B and C, the enzymes are hydrophobic surfaces (where hydrophilic regions are coloured in turquoise and hydrophobic are in tan). Resolved glycan residues (as well as how many were in the supplied glycan) are listed in B. Dextran-based ligand is shown in magenta, and the *Tp*^dex^ ligand is shown in lime green. Catalytic residues (or alanine or glycine point mutations) are shown in yellow and labelled. Waters are shown as red spheres. **E-F.** Crystallographic molecules B and A in the *Tp*^dex^ structure show different conformations of the side-wall Trp376 residue. In molecule B, W376 is in the side wall conformation, whereas in molecule A W376 is bent into the binding site, occluding subsite +1. This movement may reflect a mechanism where hydrolysis products are ejected from the enzyme [2].

**Figure S4. A comparison of surface-exposed glycoside hydrolases from the dextran (GH^dex^), levan (GH^lev^) and starch PULs (SusG, GH^starch^) of *B. theta*.** The GH_dex_ crystal structures are the wild-type (PDB ID: 9SJG) and the E360A variant with bound IMO3 (PDB ID: 9SJF). Apo structures of GH^lev^ [3] (PDB ID: 3K8K) and SusG [4] (PDB ID: 6R3R) are also included. Structures are coloured in rainbow format from N- (blue) to C- (red) terminus.

**Figure S5. GH^dex^ Asp171 stabilises substrate subsite -2 in both crystal and cryo-EM structures.** Hydrogen bonds to the glycan are shown with yellow dotted lines, and protein residues with bonding side chains are labelled and have their atoms displayed. Catalytic residues D297 (or D297A in the utilisome structure) and E360A are coloured in grey. GH^dex^ crystal structures include both the E360A variant without substrate (PDB ID: 9SJH), and with bound IMO3 (PDB ID: 9SJF). The cryo-EM structure of the closed-closed dextran utilisome is also included, with an IMO6 substrate (PDB ID: 9SM2).

**Figure S6. Comparison of Alphafold2 models and experimental structures of SGBP^dex^. A.** the full length AlphaFold model and **B.** the crystal structure of the N-terminally truncated SGBP^dex^ Δ1-147. The protein models are coloured according to their domains, including an N-terminal signal sequence that is cleaved from the final protein (dark blue), the Ig-like N-terminal domain (light blue), and three carbohydrate binding modules (CBMs; α red, β lime green and γ yellow). The first structured domain in the wild type protein (Ig-like) has been removed in the Δ1-147 truncated construct, since this domain is not predicted to have a substrate binding function. The other three CBM domains are predicted carbohydrate binding modules based on comparison with studies on the homolog SusF [5]. In the crystal structure for SGBP^dex^ Δ1-147, the linking residues between CBMα and CBMβ angle CBMα towards the viewer, whereas in the Alphafold model CBMα is angled into the background (see boxed inset panel B for a comparison). However, when aligning the CBMs of the Alphafold and crystal structure separately, CBMα and combined CBMβ-γ give Cα-RMSD values of 0.8 and 0.6 Å respectively.

**Figure S7. A non-proline *cis* peptide on the surface of SGBP^dex^ CBM**β **may contribute to its putative dextran binding site.** **A.** The crystal structure of SGBP^dex^ (Δ1-147) with the labelled *cis*-nonPro on the surface of carbohydrate binding module 2 (CBMβ). **B-C**. SusF (PDB ID: 4FE9) and SGBP^dex^ crystal structures were overlaid and then tiled to show similar regions side by side; top views are in panel B and side views in panel C. The starch ligand that binds to SusF CBMβ interacts with two aromatic surface residues (Trp287, Trp330), which resemble the putative dextran binding site on CBMβ (Trp281, Phe318, Trp304). The nearby Asp307 involved in the *cis*-nonPro peptide bond may help stabilise bound dextran through hydrogen bonding. **D.** Electron density of the A306-D307 *cis*-nonPro peptide bond (2Fo-Fc map at 1.5 σ).

**Figure S8. Comparison of SusD binding sites.** Overall views (A) of the crystal structures of SusD^dex^ (PDB ID: 9SJJ), SusD^starch^ (PDB ID: 3CK9), SusD^lev^ (PDB ID: 6Z9A) with bound substrate. Arrows point to the bound glycan. As SusD^lev^ was crystallised as a SusCD complex; SusC^lev^ was hidden from view, and *ChimeraX* matchmaker was used to align the structures. Binding sites were also shown in detail for SusD^dex^ (B), SusD^starch^ (C) and SusD^lev^ (D). Ordered waters are displayed as red spheres. Aromatic residues near the ligands are labelled. The proteins are viewed towards their ligand binding regions (Figure 3B-C). SusD^dex^ is shown as a translucent surface coloured by hydrophobicity where turquoise is hydrophilic, white is neutral and tan is hydrophobic. The resolved IMO5 is shown as sticks coloured in magenta with red representing oxygens. Glucose residues of SusD^dex^ substrate are labelled according to subsite numbering determined by cryo-EM (Figure 6). Starch and levan oligosaccharides are coloured yellow and dark green respectively. The bound ligand in SusD^starch^ is maltoheptaose (7-mer). The position of the central aromatic residue SusD^dex^ Trp284 resembles SusC^lev^ Phe649, which is provided by the SusC^lev^ loop 7.

**Figure S9. Superose 6 SEC profile and SDS-PAGE gel of the purified dextran utilisome used for cryo-EM.** "p" denotes the SEC peak fractions that were pooled.

**Figure S10. Processing diagram (pipeline 1) for the closed-closed (CC) and open-closed (OC) dextran utilisome.** Maps marked with hammers were used for early-stage model building. Arrows pointing to maps indicate the SusD^dex^ in motion. Circled maps indicate the presence of a GH^dex^ subunit. Final maps were produced through a separate processing of the same dataset (Figure S11; pipeline 2). The consensus reconstruction of pipeline 1 (~273,000 particles) was used for 3D variability which generated Movie S1 and the frames in Figure S8.

**Figure S11. Processing diagram (pipeline 2) for generation of the final closed-closed (CC) and open-closed (OC) dextran utilisome maps.** The arrow on ab-initio class 4 indicates the open SusD^dex^ lid. Top views of the maps at different processing stages **(A-D)** are shown on the right in boxes; OC is in tan and CC is in green. Maps in panel C (marked with hammers) were used for final model building. Map D was generated using multiple classes that appeared to be fully open, but due to poorer GH^dex^ density this was not used.

**Figure S12. SGBP^dex^ density in the OC and CC dextran utilisome maps. A.** Side overview comparison of the open (left) and closed (right) states. The maps are viewed from inside the utilisome (*i.e.* from the symmetry related copies of the complex) and are both contoured at 2.5 standard deviations from the mean. Although not shown in this Figure, when taking both copies of SGBP^dex^ into account, more density of the subunit is visible in the OC map. SusC^dex^ is blue, SusD^dex^ is orange-red, SGBP^dex^ NTD (1-152) is forest green, GH^dex^ is yellow and its dextran substrate is magenta. **B.** Front and back views of SGBP^dex^ CBMα (light green) and SGBP^dex^ CBMβ-γ (cyan) docked into the OC map. For simplicity, the closed copy of SusC^dex^ and its lipoproteins are not shown. An Alphafold2 [6] model of full-length SGBP^dex^ was split into two domains; SGBP^dex^ 153-261 (CBMα) and SGBP^dex^ 263-504 (CBMβ-γ). These were fit into the map in plausible orientations with respect to the N- and C-terminus and the density of the OC map. **C.** Close-up views of the SGBP^dex^:GH^dex^:SusD^dex^ interface (left) and the docked location of SGBP^dex^ subunits (right). The map is displayed within a 5 Å radius from docked SGBP^dex^ CBM domains. The OC SGBP^dex^ density points towards the GH^dex^ (yellow) binding site. In the right panel, pairs of surface-exposed aromatic residues corresponding to putative binding sites A, B and C are shown as sticks and coloured orange. The *cis* non-Pro residues Ala306-Asp307 in CBMβ are also displayed.

**Figure S13. Conformational changes during the transition between the open and closed dextran utilisome.** For simplicity, only one four-component complex (GH^dex^:SGBP^dex^:SusD^dex^:SusC^dex^) of the two that make up the full utilisome is shown. Dextran ligands have been hidden from view. **A.** The top panels show open and closed models where SusD^dex^ is hinging outwards towards the observer. The bottom panels show a side-rotated view along the rotation axis (yellow tube). Open SusD^dex^ is magenta, and closed SusD^dex^ is orange-red. *ChimeraX* “measure rotation” function was used to compare open and closed SusD^dex^. **B.** Two extracellular hinging loops (EHL) of SusC^dex^, coloured matching their SusD^dex^, contact SusD^dex^ and undergo a conformational change between open and closed utilisome states. **C-D.** Overlaid open and closed SusCD^dex^, with SusD^dex^ shown as a surface and SusC^dex^ as a cartoon. EHL1 (SusC^dex^ 627-667) and EHL2 (SusC^dex^ 697-722) are shown in full colour, while other parts of the complex are translucent. Gly647 and Thr708, at the tip of EHL1 and 2 respectively, are used to measure the movement of each loop between the open and closed states. **E.** Salt bridges (yellow dotted lines) between EHL2 residue SusC^dex^ Asp713 and SusD^dex^ Arg106, likely stabilise the CC state. Arg106 also forms a hydrogen bond with subsite 2 of the SusD^dex^ bound dextran.

**Figure S14. Comparison of SusCD "pedal bin" apertures**. All SusC homologues were aligned on SusC^lev^ (PDB ID: 6ZLT). **A.** Partly-open SusCD^lev^ (PDB ID: 6ZLT) and closed SusCD^lev^ (PDB ID: 6ZLU). SusD is brown while SusC is tan. **(B,** this study**)** Fully-open SusCD^dex^ and closed SusCD^dex^. SusD is orange red while SusC is cornflower blue. **C-D.** The open states of ButCD, a ubiquitin-homologue importer from *Bacteroides fragilis* (**C**, PDB ID: 8YPU) and RagAB, a peptide importer from *Porphyromonas gingivalis* (**D**, PDB ID: 6SML). The SusD homologues are green and cyan, while the SusC homologues are yellow and purple.

**Figure S15. SusCD^dex^ salt bridges in the closed and open states.** SusC^dex^-SusD^dex^ salt bridges were identified by the PDBePISA webserver [7] (Spreadsheet S1) and then displayed in *ChimeraX* with side chains shown. SusD^dex^ and SusC^dex^ residues are labelled in red and blue, respectively. The Asp97-Arg833 interaction (marked by asterisks) was included in the PISA analysis but *ChimeraX* did not corroborate this with the “H-bonds” function when finding salt bridges on default settings.

**Figure S16. Model-in-map views of the closed-closed (left) and open-closed (right) cryo-EM maps. A-B.** Models in transparent maps and the regions of the IMO ligand density. These maps were zoned to 5 Å around the whole model (A) or the ligand density (B). **C.** A magnified view of the ligands from the same view as (A/B), as well as a 90-degree rotation resulting in an extracellular view, where GH^dex^-associated IMO are in the foreground and the SusC^dex^ plug domain-associated IMO are in the background. IMOs are labelled with the colour of the subunit to which they are associated.

**Figure S17. The CC (A) and OC (B) dextran utilisome; Fourier-shell correlation curves, local resolution maps and maps coloured by modelled subunit.** The global resolution of the maps was estimated using the typical refinement FSC cutoff of 0.143. Local resolution was estimated in *CryoSPARC*, using a FSC cutoff of 0.5, and mapped from 2 to 6 Å ranging from blue to red.

**Figure S18. The SusCD^dex^ cavity of the CC model loaded with IMO at decreasing contours.** The CC map was coloured cyan, and then coloured by protein subunit (radius around model = 5 Å, the plug domain is orange, SusC^dex^ is blue, IMO are magenta, and SusD^dex^ is orange red). The lowest contour is also shown as opaque density (without the model) for clarity. In the first panel, terminal subsites of the IMO are numbered according to the CC model (Figure 6B). In the first and last panels, the termini of the SusD^dex^–associated IMO8 (A, B) and the SusC^dex^–associated IMO4 (C, D), as well as the non-reducing (NR) and reducing (R) ends, are labelled.

**Figure S19. A close-up view of the bottom of the aromatic lock in the dextran and levan utilisomes.** This figure shows the interface between the β-barrel wall (blue), the plug domain (orange) and the N-terminal loop (cyan). Overlaid structures from the open and closed utilisomes are included. The closed-state residues are coloured in lime green, and the open-state are coloured in purple. This view includes a stick representation of Ile218 (and Ile190 in SusC^lev^) which is not shown in Figure 7, to illustrate the contribution of the plug Ile.

**Figure S20. SusC^dex^ N-terminus disordering after SusD^dex^ lid closure.** The N-termini of open and closed SusC^dex^ are compared, where the plug domain (residues 129-232) is orange and the N-terminal loop (109-128) is cyan. The TonB box residues (^110^DEVVVV^115^), predicted by homology with other SusCs, are in grey. Tyr117, involved in the aromatic lock (Figure 7), is in purple. The first residue resolved in both open and closed SusC^dex^ (Thr119) is coloured red, with a dashed arrow showing the difference in position (16 Å) between the two models. In the open model, the first resolved residue is also labelled (Leu109).

**Table S1. Data collection and refinement statistics for crystal structures of soluble constructs of the dextran utilisome surface lipoproteins.** Values in parentheses describe the last (highest resolution) shell.

| **Protein** | **GH^dex^** | | | **SGBP^dex^** | **SusD^dex^** | |
| --- | --- | --- | --- | --- | --- | --- |
| **Mutant, ligand** | **WT, apo** | **E360A, apo** | **E360A, +IMO3** | **Δ1-147, apo** | **WT, apo** | **WT, +IMO5** |
| **PDB** | 9SJG | 9SJH | 9SJF | 9SJI | 9SJK | 9SJJ |
|  | **Data collection** | | | | | |
| **Wavelength (Å)** | 0.898 | 0.979 | 0.898 | 0.898 | 0.898 | 0.98 |
| **Space group** | P 41 21 2 | P 41 21 2 | P 41 21 2 | C 1 2 1 | P 42 21 2 | I 2 2 2 |
| **Unit cell lengths a, b, c (Å)** | 93.70, 93.70, 331.61 | 92.86, 92.86, 330.68 | 92.22, 92.22, 328.03 | 148.32, 37.55, 65.62 | 119.50, 119.50, 176.98 | 87.00, 106.15, 138.34 |
| **Unit cell angles α, β, γ (°)** | 90, 90, 90 | 90, 90, 90 | 90, 90, 90 | 90, 100.35, 90 | 90, 90, 90 | 90, 90, 90 |
| **Resolution range** | 55.27 - 1.83  (1.83-1.80) | 330.68 - 2.10  (2.14-2.10) | 47.03 - 2.20  (2.25-2.20) | 36.48 - 1.70  (1.73-1.70) | 53.44 - 1.65  (1.68-1.65) | 67.29 - 1.70  (1.73-1.70) |
| **Total reflections** | 3455703  (183119) | 2217718  (110301) | 1816516  (60863) | 270053  (14440) | 7815948  (383132) | 1786941  (101033) |
| **Unique reflections** | 137723  (6704) | 85740  (4460) | 73184  (4453) | 39600  (2069) | 153600  (7518) | 70548  (3690) |
| **Multiplicity** | 25.1  (27.3) | 25.90  (24.70) | 24.80  (13.70) | 6.80  (7.00) | 50.9  (51.0) | 25.3  (27.4) |
| **Completeness (%)** | 100.00  (100.00) | 100.0  (100.0) | 100.00  (100.00) | 100.00  (100.00) | 100.00  (100.00) | 100.00  (100.00) |
| **mean(I) / σ(I)** | 13.40  (1.6) | 5.90  (1.20) | 7.70  (1.10) | 8.00  (0.70) | 19.3  (1.0) | 19.4  (1.3) |
| **Wilson B-factor** | 22.38 | 31.57 | 30.24 | 30.63 | 23.44 | 28.02 |
| **R_merge_** | 0.16  (2.27) | 0.90  (17.35) | 0.33  (2.32) | 0.09  (2.23) | 0.15  (4.26) | 0.10  (3.09) |
| **R_meas_** | 0.17  (2.31) | 0.92  (17.72) | 0.33  (2.40) | 0.10  (2.40) | 0.15  (4.31) | 0.10  (3.14) |
| **R_pim_** | 0.03  (0.44) | 0.18  (3.58) | 0.07  (0.63) | 0.04  (0.90) | 0.02  (0.60) | 0.02  (0.60) |
| **CC_half_** | 100.0  (81.0) | 99.0  (70.0) | 100.0  (51.0) | 100.0  (71.0) | 100.0  (83.0) | 100.0  (59.0) |
|  | **Refinement** | | | | | |
| **Reflections in refinement** | 137436 | 85542 | 73027 | 39575 | 153513 | 70548 |
| **Reflections in free set** | 6909 | 4260 | 3603 | 2011 | 7727 | 3544 |
| **R_work_** | 0.198 | 0.190 | 0.202 | 0.200 | 0.180 | 0.165 |
| **R_free_** | 0.229 | 0.230 | 0.262 | 0.270 | 0.206 | 0.191 |
| **FSC average** | 0.9695 | 0.9653 | 0.9623 | 0.9341 | 0.9763 | 0.9784 |
| **RMSD bonds** | 0.0094 | 0.0067 | 0.0083 | 0.0126 | 0.012 | 0.0135 |
| **RMSD angles** | 1.8150 | 1.791 | 1.885 | 2.214 | 2.077 | 2.075 |
| **Ramachandran favoured (%)** | 97.2 | 96.5 | 97.3 | 97.4 | 98.1 | 97 |
| **Ramachandran allowed (%)** | 2.8 | 3.5 | 2.7 | 2.6 | 1.8 | 2.6 |
| **Ramachandran outliers (%)** | 0 | 0 | 0 | 0 | 0.1 | 0.4 |
| **Rotamer outliers (%)** | 1.1 | 2.9 | 2.7 | 5.1 | 1 | 1.3 |
| **Clash score** | 10.4 | 12.9 | 7 | 8 | 1.6 | 3.5 |
| **MolProbity score** | 1.73 | 2.21 | 1.86 | 2.09 | 0.92 | 1.41 |

**Table S2. Characterisation of structure-based binding site variants of GH^dex^, SGBP^dex^ and SusD^dex^ via ITC.** The fold change (to the nearest integer) decrease in *K_d_* was compared to the “wild-type” (WT) construct, described in brackets in the protein name, where each construct’s protein concentration was fixed at its calculated concentration (see Materials and methods). Ligand concentration was also fixed to the calculated value, so the wild-type values are slightly different from Table S4. Standard deviations (±) are shown in parentheses. Number of replicates refers to how many independent titrations were analysed. Titrations with no quantifiable binding are marked with NB. Example titrations are shown in Spreadsheet S2.

| **Protein** | **Dextran substrate (kDa)** | **Calculated dextran concentration (µM)** | **WT number of replicates** | **WT *K_d_***  **(µM)** | **Variant number of replicates** | **Variant *K_d_***  **(µM)** | **Decrease in *K_d_* relative to WT (fold change)** |
| --- | --- | --- | --- | --- | --- | --- | --- |
| **(GH^dex^ D297A, E360A) W260A** | 1.5 | 1600 | 3 | 1.2  (±0.2) | 2 | 76.5  (±17.3) | 62 |
| **(SGBP^dex^)**  **W394A** | 500 | 12.5 | 3 | 2.8  (±0.4) | 3 | 13.2  (±4.4) | 5 |
| **(SGBP^dex^)**  **W433A** | 500 | 12.5 |  |  | 3 | 15.1  (±1.2) | 5 |
| **(SusD^dex^)**  **Y103A** | 1.5 | 1600 | 3 | 13.7 (±2.5) | 3 | 127.2  (±37.9) | 9 |
| **(SusD^dex^)**  **W284A** | 1.5 | 1600 |  |  | 3 | NB | NB |
| **(SusD^dex^)**  **F296A** | 1.5 | 1600 |  |  | 2 | NB | NB |

**Table S3. Data collection, map and real-space refinement statistics from cryo-EM of the dextran utilisome in the closed-closed (CC) and open-closed (OC) states**. Map resolution was estimated by the non-uniform refinement job in *CryoSPARC* (after FSC-mask auto-tightening and using a 0.143 cut-off) and supplied to *Phenix* for real-space refinement. Local resolution was estimated using the *CryoSPARC* job which implements a local windowed FSC method [8]. Models were refined using the final, octameric maps (see Figure S11C).

| **Data collection** | | | | |
| --- | --- | --- | --- | --- |
| **Category** | **Metric** | **OC**  **EMD-54945** | **CC**  **EMD-55024** | |
|  | **Number of movies** | 12,408 | | |
|  | **Magnification** | 105,000 | | |
|  | **Defocus range (µm)** | -1.0 to -2.0 | | |
|  | **Microscope** | TFS Krios | | |
|  | **Voltage (kV)** | 300 | | |
|  | **Electron dose (e^-^/Å^2^)** | 23.1 | | |
|  | **Image detector** | Gatan K3 | | |
| **Processing** | | | | |
| **Number of particles** | **Picked** | 4,556,980 | | |
|  | **After 2D classification** | 481,934 | | |
|  | **Final 3D refinement** | 23,484 | 116,104 | |
|  | **Recommended contour level** | 0.025 | 0.015 | |
|  | **Symmetry imposed** | C1 | C1 | |
|  | **Pixel spacing (Å/pixel)** | 0.831 | | |
|  | **Map resolution (Å)**  **(FSC threshold 0.143)** | 2.9 | | 2.49 |
| **Local resolution estimation (Å)**  **(FSC threshold 0.5)** | **Range** | 1.78 to 48.49 | 2.19 to 39.53 | |
|  | **25^th^ percentile** | 3.76 | 2.81 | |
|  | **Median** | 6.92 | 4.35 | |
|  | **75^th^ percentile** | 8.35 | 6.14 | |
| **Refinement** | | | | |
| **Category** | **Metric** | **OC** | **CC** | |
| **PDB** |  | **9SJE** | **9SM2** | |
| **Composition (#)** | **Chains** | 11 | 11 | |
|  | **Atoms** | 32922 (Hydrogens: 0) | 32876 (Hydrogens: 0) | |
|  | **Residues** | Protein: 4131 Nucleotide: 0 | Protein: 4124 Nucleotide: 0 | |
|  | **Water** | 0 | 0 | |
|  | **Ligands** | GLC: 35 | GLC: 36 | |
| **Bonds (RMSD)** | **Length (Å) (# > 4σ)** | 0.004 (0) | 0.003 (2) | |
|  | **Angles (°) (# > 4σ)** | 0.816 (12) | 0.691 (13) | |
| **MolProbity** | **MolProbity score** | 1.56 | 1.37 | |
|  | **Clash score** | 1.99 | 1.79 | |
| **Ramachandran plot (%)** | **Outliers** | 0.70 | 0.58 | |
|  | **Allowed** | 4.50 | 4.02 | |
|  | **Favored** | 94.80 | 95.40 | |
| **Rama-Z** | **Whole (N = 4115 / 4108)** | -0.19 (0.13) | 0.14 (0.13) | |
|  | **Helix** | -1.21 (0.15) | -0.62 (0.16) | |
|  | **Sheet** | 1.04 (0.15) | 1.21 (0.15) | |
|  | **Loop** | -0.23 (0.14) | -0.13 (0.14) | |
| **Side Chain Quality** | **Rotamer outliers (%)** | 2.07 | 1.41 | |
|  | **Cß outliers (%)** | 0.00 | 0.00 | |
| **Peptide Plane (%)** | ***Cis* proline / general** | 1.4 / 0.0 | 1.4 / 0.0 | |
|  | **Twisted proline / general** | 0.0 / 0.0 | 0.0 / 0.0 | |
| **CaBLAM outliers (%)** |  | 2.83 | 2.17 | |
| **ADP (B-factors)** | **Iso/Aniso (#)** | 32922 / 0 | 32876 / 0 | |
|  | **Protein (min/max/mean)** | 14.39 / 236.92 / 106.65 | 28.15 / 248.56 / 102.54 | |
|  | **Ligand (min/max/mean)** | 20.00 / 166.78 / 98.05 | 20.00 / 147.15 / 90.05 | |
| **Occupancy** | **Mean** | 1.00 | 1.00 | |
|  | **occ = 1 (%)** | 100.00 | 100.00 | |
|  | **0 < occ < 1 (%)** | 0.00 | 0.00 | |
|  | **occ > 1 (%)** | 0.00 | 0.00 | |
| **Box Dimensions (Å)** | **Lengths** | 152.90, 174.51, 177.00 | 154.57, 178.66, 181.16 | |
|  | **Angles** | 90.00, 90.00, 90.00 | 90.00, 90.00, 90.00 | |
| **Resolution (Å)** | **Supplied** | 2.9 | 2.5 | |
|  | **d99 (full/half1/half2)** | 3.5 / --- / --- | 3.1 / --- / --- | |
|  | **d model** | 3.3 | 3.0 | |
|  | **d FSC model (0/0.143/0.5)** | 2.8 / 2.9 / 3.2 | 2.5 / 2.6 / 3.1 | |
| **Map** | **min / max / mean** | -0.09 / 0.19 / 0.00 | -0.07 / 0.19 / 0.00 | |
| **Model vs. Data** | **CC (mask)** | 0.88 | 0.87 | |
| **(Cross-correlation)** | **CC (box)** | 0.76 | 0.77 | |
|  | **CC (peaks)** | 0.66 | 0.66 | |
|  | **CC (volume)** | 0.87 | 0.87 | |
|  | **Mean CC for ligands** | 0.72 | 0.81 | |

**Table S4. Binding affinity (*K_d_*) for purified recombinant dextran utilisome surface lipoproteins when titrated with dextrans ranging from 1.5 to 500 kDa.** This analysis was performed by fixing the protein concentration to the calculated amount and *n* (ITC parameter for stoichiometry) to 1, while allowing the software to change the dextran concentration from the calculated value to fit the data (the expected values given to the software are listed in column 3). To represent the variation in each set of replicates, standard deviations (±) are shown. Example titrations are shown in Spreadsheet S2.

| **Protein** | **Dextran substrate (kDa)** | **Calculated dextran concentration (µM)** | **Number of replicates (N)** | ***K_d_***  **(µM)** |
| --- | --- | --- | --- | --- |
| **GH^dex^ E360A** | **1.5** | 1600 | 3 | 2.54 (±0.22) |
|  | **3.5** | 800 | 3 | 1.29 (±0.13) |
|  | **10** | 250 | 3 | 2.81 (±0.04) |
|  | **20** | 100 | 3 | 2.11 (±0.26) |
|  | **40** | 50 | 5 | 3.51 (±1.16) |
|  | **150** | 25 | 3 | 2.84 (±0.73) |
|  | **500** | 12.5 | 3 | 2.33 (±1.32) |
| **GH^dex^ D297A, E360A** | **1.5** | 1600 | 3 | 2.01 (±0.58) |
|  | **3.5** | 800 | 3 | 3.01 (±0.33) |
|  | **500** | 12.5 | 3 | 3.72 (±1.58) |
| **SGBP^dex^** | **1.5** | 1600 | 5 | 6603.24 (±9039.12) |
|  | **3.5** | 800 | 3 | 25.97 (±2.25) |
|  | **10** | 400 | 3 | 16.23 (±0.97) |
|  | **20** | 400 | 3 | 8.43 (±0.20) |
|  | **40** | 400 | 3 | 6.55 (±0.86) |
|  | **150** | 25, 30 | 3 | 6.10 (±1.22) |
|  | **500** | 12.5 | 3 | 2.76 (±0.45) |
| **SGBP^dex^ Δ1-147** | **1.5** | 1600 | 3 | 101.90 (±14.9) |
|  | **3.5** | 800 | 3 | 32.83 (±3.73) |
|  | **10** | 800 | 3 | 20.07 (±2.66) |
|  | **20** | 200 | 3 | 11.57 (±0.65) |
|  | **40** | 100 | 3 | 6.71 (±0.76) |
|  | **150** | 50 | 3 | 3.66 (±0.58) |
|  | **500** | 10, 12.5 | 3 | 3.23 (±2.49) |
| **SusD^dex^** | **1.5** | 1600 | 3 | 15.57 (±1.92) |
|  | **3.5** | 800 | 3 | 12.56 (±4.01) |
|  | **10** | 250 | 3 | 13.77 (±0.06) |
|  | **20** | 100 | 3 | 7.69 (±1.33) |
|  | **40** | 50 | 3 | 14.70 (±2.63) |
|  | **150** | 25 | 3 | 15.53 (±2.57) |
|  | **500** | 12.5 | 3 | 18.70 (±0.70) |

**Table S5. Stoichiometry analysis of dextran binding proteins.** Using a one set of sites binding model, the *n* value (number of binding sites) was allowed to vary to fit the data while keeping the supplied dextran concentration fixed. This is different from the previous analysis (Table S4), where *n* was fixed to equal 1 and the dextran concentration was varied. Three independent runs were analysed using this method and the *n* values were then averaged. Values marked with asterisks were deemed untrustworthy due to the software’s modelling function hitting a lower bound for ΔH and these values were not averaged. Standard deviations are reported in brackets.

| **Dextran binding protein** | **Dextran 1.5 (*n*)** | **Mean** | **Dextran 3.5 (*n*)** | **Mean** |
| --- | --- | --- | --- | --- |
| **GH^dex^ E360A** | 1.98, 2.25, 2.13 | 2.12 (±0.14) | 0.920, 0.934, 0.976 | 0.94 (±0.03) |
| **SGBP^dex^** | 3.79*, 0.606, 0.336*, 0.483* | N/A | 1.26, 1.26, 1.17 | 1.23 (±0.05) |
| **SGBP^dex^ Δ1-147** | 1.83, 1.36, 1.34 | 1.51 (±0.28) | 0.556*, 0.758, 1.36 | N/A |
| **SusD^dex^** | 1.01, 1.03, 1.00 | 1.01 (±0.02) | 0.492, 0.507, 0.463 | 0.49 (±0.02) |

**Table S6. Descriptions of Movies S1 and S2.**

| **Movie** | **Description** |
| --- | --- |
| Movie S1. The closing of SusD^dex^ and SGBP^dex^ supplying  glycans to GH^dex^ | An accompaniment to Figure 5. A 20-frame movie was generated by *CryoSPARC* 3D variability on a consensus stack of ~270k particles (Figure S10). This movie shows the SusD^dex^ in motion from its open to closed state. The C-terminal domain (CTD) of SGBP^dex^, which is poorly resolved in other 3D reconstructions, is observed at the beginning and the end of this motion. At the beginning, the CTD contacts the GH^dex^ binding site and a bridge of density is observed (44-45s); this may reflect how glycans are passed from SGBP^dex^ to GH^dex^. As the SusD^dex^ closes, SGBP^dex^ simultaneously shifts away from GH^dex^ and appears to be more resolved resting upon the top of closed SusD^dex^ (43-44s). |
| Movie S2. Dextran utilisome binding sites in the closed-closed model. | An accompaniment to Figure 6. Movie generated in *ChimeraX*. Binding sites for GH^dex^, SusD^dex^ and SusC^dex^ are shown in sequence, with bound IMO6, IMO8 and IMO4 (dextran ligands), respectively. Residues within 5Å of each dextran ligand are displayed as grey sticks, while IMO are displayed in magenta. SusC^dex^ is cornflower blue, while its plug domain (residues 129-232) is coloured orange. SusD^dex^ is orange-red, GH^dex^ is yellow and SGBP^dex^ is forest green. Only the N-terminal domain of SGBP^dex^ (non-carbohydrate-binding, Ig-like) was resolved sufficiently for modelling. |

**Supplementary discussion**

**SGBP^dex^ Δ1-147 crystal structure and its CBMβ *cis* peptide bond.**

In the crystal structure of SGBP^dex^ Δ1-147, a loop at the top surface of CBMβ contains a *cis* peptide bond between Ala306 and Asp307 (Figure S7), which is rare for bonds that do not contain prolines (*cis* non-Pro) [9]. This bond is well supported by electron density (Figure S7D). *Cis* non-Pro motifs have been identified to be important for biological activity, including ligand binding, dimerisation and catalysis [10–12]. Genuine *cis* non-Pro bonds are also relatively more common in carbohydrate-active proteins, in which they are overrepresented compared to non-carbohydrate binding proteins [12]. In SGBP^dex^ Δ1-147, conformational restriction by an upstream proline (Pro303) may influence this bonding. There are reports of downstream prolines reducing the barrier to *cis*/*trans* rotation in the peptide preceding the proline [12,13], but it is unclear if upstream prolines also have an effect. Ala307/Asp308 is surrounded by candidate surface-exposed dextran-binding residues including an angled surface of Trp281-Phe318 and Trp304. Somewhat similarly, in SusF, CBMB (equivalent to CBMβ in SGBP^dex^) residues Trp287 and Trp330 form an aromatic platform that binds starch, behind which Thr289-Pro290 form a *cis*-Pro peptide bond [5]. The SGBP^dex^ Δ1-147 *cis* peptide between Ala306-Asp307 may support dextran binding by projecting Asp307 towards a glycan cooperatively bound by the proximal Trp281-Phe318 aromatic surface residues, which are spatially analogous to confirmed binding residues Trp287 and Trp330 in SusF CBMB. Asp307 may stabilise bound dextran by hydrogen bonding. Additionally, dextran binding to CBMβ could instigate *cis*-to-*trans* isomerisation, driving the movement of SGBP^dex^ towards GH^dex^ seen in cryo-EM (Figure 5, Movie S1). In Figure S12C, the density shown of an open utilisome around the estimated position of binding site β in CBMβ is particularly weak, perhaps suggesting that this is the pivot point of SGBP^dex^ movement. This region also contains the *cis* Ala306-Asp307. There are also other examples of functional isomerisation of *cis* non-Pro peptide bonds. Concanavalin A, a lectin, is known to be in equilibrium between locked and unlocked conformational states. Based on its crystal structure, this was proposed to be caused by the binding of a metal ion, which drives a *trans*-to-*cis* isomerisation of an Ala-Asp peptide bond [14]. Binding of the glycan methyl-α-D-mannose alone was also observed to lock metal-free concanavalin A via peptide bond isomerisation [14].

**References**

[1]	Y.-M. Kim, Y. Kiso, T. Muraki, M.-S. Kang, H. Nakai, W. Saburi, W. Lang, H.-K. Kang, M. Okuyama, H. Mori, R. Suzuki, K. Funane, N. Suzuki, M. Momma, Z. Fujimoto, T. Oguma, M. Kobayashi, D. Kim, A. Kimura, Novel dextranase catalyzing cycloisomaltooligosaccharide formation and identification of catalytic amino acids and their functions using chemical rescue approach, J. Biol. Chem. 287 (2012) 19927–19935. https://doi.org/10.1074/jbc.M111.339036.

[2]	N. Suzuki, N. Kishine, Z. Fujimoto, M. Sakurai, M. Momma, J.-A. Ko, S.-H. Nam, A. Kimura, Y.-M. Kim, Crystal structure of thermophilic dextranase from Thermoanaerobacter pseudethanolicus, J. Biochem. 159 (2016) 331–339. https://doi.org/10.1093/jb/mvv104.

[3]	K. Ernits, P. Eek, T. Lukk, T. Visnapuu, T. Alamäe, First crystal structure of an endo-levanase – the BT1760 from a human gut commensal *Bacteroides thetaiotaomicron*, Sci. Rep. 9 (2019) 8443. https://doi.org/10.1038/s41598-019-44785-0.

[4]	N.M. Koropatkin, T.J. Smith, SusG: A Unique Cell-Membrane-Associated α-Amylase from a Prominent Human Gut Symbiont Targets Complex Starch Molecules, Structure 18 (2010) 200–215. https://doi.org/10.1016/j.str.2009.12.010.

[5]	E.A. Cameron, M.A. Maynard, C.J. Smith, T.J. Smith, N.M. Koropatkin, E.C. Martens, Multidomain Carbohydrate-binding Proteins Involved in *Bacteroides thetaiotaomicron* Starch Metabolism, J. Biol. Chem. 287 (2012) 34614–34625. https://doi.org/10.1074/jbc.M112.397380.

[6]	J. Jumper, R. Evans, A. Pritzel, T. Green, M. Figurnov, O. Ronneberger, K. Tunyasuvunakool, R. Bates, A. Žídek, A. Potapenko, A. Bridgland, C. Meyer, S.A.A. Kohl, A.J. Ballard, A. Cowie, B. Romera-Paredes, S. Nikolov, R. Jain, J. Adler, T. Back, S. Petersen, D. Reiman, E. Clancy, M. Zielinski, M. Steinegger, M. Pacholska, T. Berghammer, S. Bodenstein, D. Silver, O. Vinyals, A.W. Senior, K. Kavukcuoglu, P. Kohli, D. Hassabis, Highly accurate protein structure prediction with AlphaFold, Nature 596 (2021) 583–589. https://doi.org/10.1038/s41586-021-03819-2.

[7]	E. Krissinel, K. Henrick, Inference of Macromolecular Assemblies from Crystalline State, J. Mol. Biol. 372 (2007) 774–797. https://doi.org/10.1016/j.jmb.2007.05.022.

[8]	G. Cardone, J.B. Heymann, A.C. Steven, One number does not fit all: Mapping local variations in resolution in cryo-EM reconstructions, J. Struct. Biol. 184 (2013) 226–236. https://doi.org/10.1016/j.jsb.2013.08.002.

[9]	A. Jabs, M.S. Weiss, R. Hilgenfeld, Non-proline Cis peptide bonds in proteins, J. Mol. Biol. 286 (1999) 291–304. https://doi.org/10.1006/jmbi.1998.2459.

[10]	K.P. Exarchos, T.P. Exarchos, G. Rigas, C. Papaloukas, D.I. Fotiadis, Extraction of consensus protein patterns in regions containing non-proline cis peptide bonds and their functional assessment, BMC Bioinformatics 12 (2011) 142. https://doi.org/10.1186/1471-2105-12-142.

[11]	B.L. Stoddard, S. Pietrokovski, Breaking up is hard to do, Nat. Struct. Biol. 5 (1998) 3–5. https://doi.org/10.1038/nsb0198-3.

[12]	J.S. Richardson, L.L. Videau, C.J. Williams, B.J. Hintze, S.M. Lewis, D.C. Richardson, *Cis* ‐ nonProline peptides: Genuine occurrences and their functional roles, Protein Sci. 34 (2025) e70157. https://doi.org/10.1002/pro.70157.

[13]	D. Pal, P. Chakrabarti, Cis peptide bonds in proteins: residues involved, their conformations, interactions and locations 1 1Edited by J. M. Thornton, J. Mol. Biol. 294 (1999) 271–288. https://doi.org/10.1006/jmbi.1999.3217.

[14]	J. Bouckaert, Y. Dewallef, F. Poortmans, L. Wyns, R. Loris, The Structural Features of Concanavalin A Governing Non-proline Peptide Isomerization, J. Biol. Chem. 275 (2000) 19778–19787. https://doi.org/10.1074/jbc.M001251200.
